# JAK Inhibitors for Treatment of Pyoderma Gangrenosum and Sweet Syndrome: A Systematic Review of Published Case Reports

**DOI:** 10.1155/drp/7086209

**Published:** 2026-07-18

**Authors:** Seyed Mohammad Vahabi, Sama Heidari, Yalda Farahmand, Elnaz Pourgholi, Nika Kianfar, Huria Memari, Farzad Esmaeili, Saeed Bahramian, Ifa Etesami, Mahshid Sadat Ansari

**Affiliations:** ^1^ Department of Dermatology, Razi Hospital, Tehran University of Medical Sciences, Tehran, Iran, tums.ac.ir; ^2^ Autoimmune Bullous Diseases Research Center, Department of Dermatology, Razi Hospital, Tehran University of Medical Sciences, Tehran, Iran, tums.ac.ir

**Keywords:** JAK inhibitors, JAK/STAT signaling pathway, neutrophilic dermatoses, pyoderma gangrenosum, sweet syndrome

## Abstract

Neutrophilic dermatoses, including pyoderma gangrenosum (PG) and Sweet syndrome (SS), are inflammatory disorders characterized by neutrophilic infiltration without infection. Conventional therapies are often inadequate. We aim to evaluate the efficacy and safety of JAK inhibitors (JAK‐I) in the treatment of PG and SS. The study was registered (PROSPERO‐CRD420251113331), and a relevant search was conducted on PubMed/MEDLINE, Scopus, Web of Science, and Embase until July 27, 2025. Included studies were English language reports describing PG or SS treated with any JAK‐I or cases of JAK‐I–associated SS. Reviews, animal studies, and reports with insufficient clinical data were excluded. Four reviewers independently screened records. Data extraction included demographics, comorbidities, treatment regimens, outcomes, and adverse events. Risk of bias was assessed using the National Heart, Lung, and Blood Institute and Murad et al. tools through discussion‐based consensus. Due to heterogeneous outcome definitions and small sample sizes, only descriptive synthesis was performed. Fifty‐four reports, including 70 patients (59 PG, 5 SS) treated with JAK‐I and 6 JAK‐I–associated SS, were included. Across 43 PG studies, five JAK‐Is—tofacitinib, upadacitinib, baricitinib, abrocitinib, and ruxolitinib—produced 33 complete and 26 partial responses. Twenty‐six patients (44.1%) received monotherapy. Most patients (51/59; 86.4%) had at least one comorbidity. Adverse events occurred in 6 (10.1%), including anemia, hypertension, renal dysfunction, fatigue, and acneiform eruption. Among 11 SS reports, all 5 treated patients achieved complete resolution with baricitinib, ruxolitinib, or filgotinib. Six additional cases described ruxolitinib‐associated SS, generally improving with corticosteroids or drug withdrawal. Most studies were rated good quality. Evidence is limited to small cases with heterogeneous outcome definitions, variable dosing, and inconsistent follow‐up. JAK‐Is are associated with clinical improvement in PG and selected SS cases and may serve as useful adjunct therapies, though caution is needed in patients with hematologic malignancies. Larger controlled studies are needed.

## 1. Introduction

Neutrophilic dermatoses (NDs) are a group of inflammatory diseases characterized by a neutrophilic infiltration in one or more skin layers on histopathology without an underlying infection. NDs include conditions such as pyoderma gangrenosum (PG) and Sweet syndrome (SS), which exist on a spectrum with or without extracutaneous manifestations. The pathogenesis of NDs is not fully elucidated, but some are linked to genetic factors [1].

PG is characterized by painful, rapidly growing skin ulcers with irregular violaceous or erythematous edges. The pathogenesis is not fully understood, but immune system and Janus kinase (JAK) mutations play a role. More than 50% of PG patients have systemic diseases, with inflammatory bowel disease (IBD) more common in younger patients and arthritis or malignancies more prevalent in older individuals. Diagnosing PG is challenging, based on various criteria, biopsy, and lesion culture. Current treatments include systemic corticosteroids, cyclosporine, methotrexate, intravenous immunoglobulin, and biological agents like antitumor necrosis factor alpha (TNF‐α) [1–3].

SS is characterized by single or multiple, rapidly evolving and painful skin lesions in a febrile patient. The clinical presentation varies from plaques or papules to cellulitis‐like or necrotizing forms. It can present as idiopathic or as an inflammatory‐related, malignancy‐related, or infection‐related condition. The pathogenesis is multifactorial and depends on disease subtype. Diagnosis is based on clinicopathological features, and treatment includes corticosteroids and anti‐TNF‐α agents [1, 4].

The JAK/STAT signaling pathway is involved in immune regulation, apoptosis, and cell proliferation and has been implicated in autoimmune and inflammatory diseases, as well as malignancies [5, 6]. By acting on four proteins (JAK1, JAK2, JAK3, and TYK2), JAK inhibitor (JAK‐I) drugs are used in various cutaneous and systemic diseases. JAK‐Is have recently been approved for rheumatoid arthritis, ulcerative colitis, alopecia areata, and atopic dermatitis, and studies suggest that they could be potential therapeutic options for vitiligo, morphea, psoriasis, and hidradenitis suppurativa [7–10].

This systematic review aims to evaluate the role of JAK‐Is in the treatment of PG and SS.

## 2. Materials and Methods

This systematic review follows the 2020 guidelines of the Preferred Reporting Items for Systematic Reviews and Meta‐analyses (PRISMA) [11]. It is registered in the PROSPERO database (CRD420251113331).

### 2.1. Search Strategy

A systematic search was conducted using keywords/MeSH terms related to SS, PG, and JAK‐Is through PubMed/Medline, Scopus, Web of Science, and Embase until July 27th, 2025 (Supporting file 1).

### 2.2. Eligibility Criteria and Study Selection

Patients with PG or SS who used at least one JAK‐I were included. Reviews, non‐English articles, articles with incomplete substantial data, and animal/in vitro studies were excluded. We also included studies that showed JAK‐I‐associated SS.

### 2.3. Data Extraction

Initially, four reviewers independently screened the articles to exclude unrelated ones. In case of disagreement, a fifth reviewer or the corresponding author made the final decision. Data extracted included study characteristics, patient age, sex, past medical history, disease duration, previous treatments, dosage, and duration of received JAK‐I(s), other concurrent medications, outcomes, and possible adverse effects.

### 2.4. Definition of Outcome

The primary outcome was defined as the clinical assessment of ulcer improvement as reported by the original authors. Because terminology varied substantially across studies, we applied standardized outcome categories for this review. A complete response was assigned only when authors explicitly described full clinical healing using terms such as “complete response,” “complete healing,” “complete clearance,” or “total resolution.” Descriptors such as “almost complete,” “nearly healed,” or other near‐complete terms were classified as partial response. Outcomes described as improvement or minor improvement were also categorized as partial response. Cases in which no clinical healing was reported or where the available information was insufficient to determine healing status were classified as no response. In one study that used a 0–4 Physician Global Assessment (PGA) scale, a score of 1 was categorized as a complete response and a score of 2 as a partial response. Adverse events were evaluated as a secondary outcome.

### 2.5. Risk of Bias

Quality assessment for included studies was done by a discussion‐based assessment between two authors (S.H. and Y.F). We used National Heart, Lung, and Blood Institute (NHLBI) quality assessment tools for a retrospective study and Murad et al.’s [12] critical appraisal tool (Supporting file 2).

### 2.6. Analysis

Statistical analysis was not applicable for this review because the included reports used heterogeneous and nonstandardized outcome measures, which precluded meaningful comparison across studies. The variability in reporting formats, small case numbers within individual JAK‐I subgroups, and inconsistent use of concomitant therapies further limited the feasibility of any inferential testing. Descriptive statistics were therefore calculated based on the available case‐level data only; when specific variables were missing in individual reports (e.g., one study of four patients did not provide sex distribution), percentages and means were derived from the remaining cases with reported information.

## 3. Results

### 3.1. Overview

**FIGURE 1:**
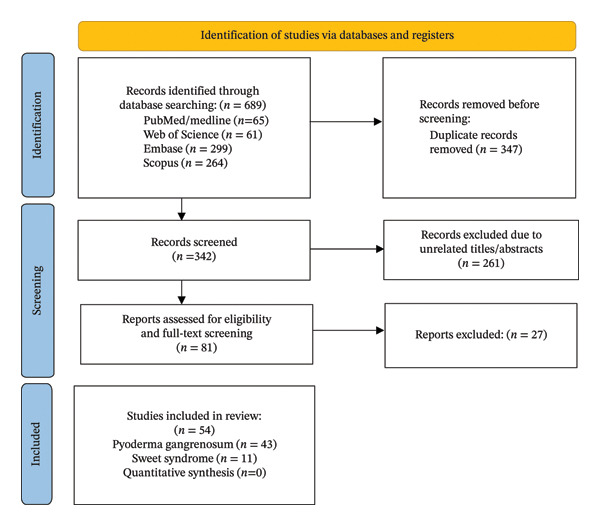
Preferred reporting items for systematic reviews and meta‐analyses (PRISMA) Flowchart of the number of studies identified and selected into the systematic review and meta‐analysis.

A total of 342 records were initially identified across all databases. After screening and eligibility assessment, 54 articles met the inclusion criteria; 43 focused on PG and 11 on SS (Figure 1).

Of these, 59 cases of PG, 5 cases of SS treated with JAK‐I, and 6 cases of JAK‐I‐associated SS were identified. Multiple JAK‐Is were used for PG, including abrocitinib, baricitinib, ruxolitinib, tofacitinib, and upadacitinib. For SS, treatment with baricitinib, ruxolitinib, or filgotinib was reported in cases. Ruxolitinib was the only JAK‐I reported to induce SS (Table 1).

**TABLE 1:** Overview of disease, JAK inhibitors, outcomes, and adverse events.

Disease	Number of patients	JAK inhibitor treatment	Outcome (response to treatment)	Adverse effects
Pyoderma gangrenosum	3	Abrocitinib	Complete (*n* = 1), Partial (*n* = 2)	Acneiform eruption (*n* = 1)
	10	Baricitinib	Complete (*n* = 4), Partial (*n* = 6)	Decreased renal function (*n* = 1)
	3	Ruxolitinib	Complete (*n* = 1), Partial (*n* = 2)	Anemia (*n* = 1)
	24	Tofacitinib	Complete (*n* = 14), Partial (*n* = 10)	Arterial hypertension (*n* = 1)
	19	Upadacitinib	Complete (*n* = 13), Partial (*n* = 6)	Fatigue and mild anemia (*n* = 2)

Sweet Syndrome treatment	2	Baricitinib	Complete response	None
	2	Ruxolitinib	Complete response	None
	1	Filgotinib	Complete response	None

JAK‐inhibitor associated Sweet Syndrome	6	Ruxolitinib	Steroids (*n* = 4) JAK‐I withdrawal (*n* = 1) No resolution (*n* = 1)	NA

### 3.2. PG

A total of 43 reports comprising 59 patients diagnosed with PG were included in this study (Supporting Table 1). Of these patients, 38 (69.1%) were female and 17 (30.9%) were male, while the sex was not reported for four individuals [13]. Age was also unspecified in five cases [13, 14]; for the remaining patients, the mean age was 52.05 ± 17.6 years.

**FIGURE 2:**
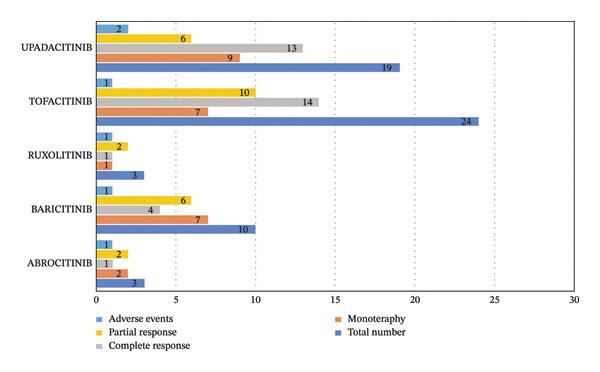
Summary of treatment outcomes and adverse events reported across JAK inhibitors for pyoderma gangrenosum treatment. Bars represent the total number of cases for each agent, categorized by complete response, partial response, monotherapy use, and adverse events.

Across the included studies, five different JAK‐I were administered: tofacitinib (*n* = 24, 40.7%), upadacitinib (*n* = 19, 32.2%), baricitinib (*n* = 10, 16.9%), abrocitinib (*n* = 3, 5.1%), and ruxolitinib (*n* = 3, 5.1%). Overall, 33 patients (55.9%) achieved a complete response to JAK‐I therapy, with a mean (±SD) time to response of 4.56 ± 3.9 months. An additional 26 patients (44.1%) experienced a partial response, with a mean (±SD) time to improvement of 2.78 ± 1.8 months. In one report [15], ulcerative lesions recurred four weeks after discontinuation of baricitinib; in another case [16], partial response to abrocitinib was observed within one month, but treatment was discontinued due to an adverse effect, which subsequently triggered PG flares. In a recent report [17], although clinical improvement was achieved, the patient died due to an underlying comorbidity (Figure 2).

The lower extremities were the most commonly affected sites (*n* = 46, 77.9%), followed by the trunk (*n* = 12, 20.3%), genitalia (*n* = 11, 18.6%), upper limbs (*n* = 9, 15.2%), and head and neck (*n* = 6, 10.1%). Thirteen cases (22%) involved more than one anatomical location. In two patients, the anatomical site was not specified in the report [13].

Twenty‐six patients (44.1%) received JAK‐I monotherapy, while the remaining patients were treated with at least one concomitant therapy. Except for two individuals, the majority (96.6%) had undergone prior treatments before initiation of JAK‐I therapy. Thirty‐three patients (55.9%) underwent biopsy, which demonstrated findings consistent with PG and helped exclude other common causes of ulceration (Figure 2).

Regarding comorbidities, 51 patients (86.4%) had at least one comorbidity, while 8 (13.6%) had none. A history of autoimmune disease was present in 44 patients (74.6%), including 14 (23.7%) with IBD and 11 (18.6%) with rheumatoid arthritis. Additionally, 6 patients (10.1%) had a history of malignancy.

#### 3.2.1. Tofacitinib

Twenty‐four individuals were treated with tofacitinib, at doses ranging from 5 mg/day up to 20 mg/day. Seventeen patients received a combination of tofacitinib and another medication [13, 14, 18–25], and seven had a monotherapy [20, 25–29]. Among all reported patients, only one adverse event was reported [22]. 14 individuals showed a complete response to treatment, and ten had a partial response. The duration of response from treatment initiation in the tofacitinib group ranged from 3 weeks to 18 months.

#### 3.2.2. Baricitinib

Ten cases were investigated for the use of baricitinib, with three cases involving its use in combination with other drugs [15, 30, 31] and seven cases as monotherapy [26, 32–36]. The dosage ranged from 2 to 4 mg daily, leading to complete response in four cases. One case was reported with an adverse effect (decreased renal function) leading to a change from baricitinib to upadacitinib for maintenance therapy [35]. Six cases had partial response, with one of them reported with recurrence after baricitinib discontinuation [15]. The duration of response in the baricitinib group ranged from two weeks to six months.

#### 3.2.3. Abrocitinib

Three patients were treated with this drug [16, 37, 38], one in combination with cyclosporine A [16] and two as monotherapy. Complete response was achieved in one case. Although one patient experienced significant improvement, abrocitinib was discontinued after one month due to an adverse effect [16].

#### 3.2.4. Upadacitinib

Nineteen patients were included in this study, of whom ten received the drug in combination with other medications [39–46], all involving corticosteroids, and nine patients were treated with upadacitinib monotherapy [41, 47–53]. Of these, 13 cases showed a complete response, while others had a partial response to the treatment. The duration of response from treatment initiation in the upadacitinib group ranged from two weeks to 8.5 months. In two patients, mild anemia, body heaviness, and fatigue were the only reported adverse effects [45, 52]. Except for three cases with an unknown dosage [42, 49, 51], the dose of upadacitinib ranged from 15 mg/day to 45 mg/day, depending on the patient’s condition.

#### 3.2.5. Ruxolitinib

There were three cases investigated using ruxolitinib for PG treatment. One of them had used ruxolitinib alone [54], and two patients had it paired with other medications [17, 55]. A complete response was observed in one case, although the patient developed anemia as an associated adverse event [54]. A partial response was observed in two cases, both treated with daily dosages of 10–40 mg [17, 55].

#### 3.2.6. Adverse Effects

Among the 59 patients who received a JAK‐Is for PG treatment, six patients (10.1%) showed adverse effects. The first patient, who was a 69‐year‐old woman, developed high blood pressure [22], which could be due to the simultaneous administration of prednisolone. Two patients treated with ruxolitinib and upadacitinib [45, 54] had shown mild anemia. One patient experienced decreased renal function during treatment, necessitating a change in the JAK‐I for maintenance therapy [35]. In another case, treatment was complicated by an acneiform eruption, prompting cessation of abrocitinib and subsequent PG exacerbation despite early therapeutic benefit [16]. Additionally, one patient reported an uncommon side effect, described as body heaviness and fatigue, which led to self‐discontinuation of the JAK‐I [52].

### 3.3. SS

Eleven individuals were studied with SS. The mean (±SD) age was 62.5 ± 10, and 6 (54.5%) were female. All the cases had positive biopsies for SS diagnosis. Three different JAK‐Is were used in the treatment of SS, including filgotinib, ruxolitinib, and baricitinib. While five patients had a significant improvement from using JAK‐Is (Supporting Table 2), the other six had shown JAK‐I‐associated SS (Supporting Table 3). Among SS patients treated with JAK‐Is, all demonstrated a complete response; however, one patient died due to progression of the underlying disease [56]. Two patients were reported with hematological malignancies, two with rheumatoid arthritis, and one with chronic gastritis.

#### 3.3.1. SS Treated With JAK‐Is

##### 3.3.1.1. Filgotinib

A 64‐year‐old woman with long‐standing rheumatoid arthritis developed acute SS with painful plaques, fever, and neutrophilic leukocytosis. She had failed multiple disease‐modifying antirheumatic drugs (DMARDs) and biologics, but filgotinib 200 mg/day induced complete resolution of cutaneous and systemic symptoms, with sustained remission [57].

##### 3.3.1.2. Baricitinib

A 59‐year‐old woman with rheumatoid arthritis–associated SS presented with erythematous edematous plaques, fever, and arthralgia. Baricitinib 2 mg/day led to complete healing within four weeks and durable remission over ten months [58].

A 50‐year‐old woman with ND of the dorsal hands, a localized SS variant, experienced painful plaques unresponsive to corticosteroids. Baricitinib 2 mg/day produced visible improvement within 24 h and complete resolution by week four, with no recurrence during one year of follow‐up [59].

##### 3.3.1.3. Ruxolitinib

A 55‐year‐old woman with monoclonal gammopathy of undetermined significance (MGUS)‐associated refractory SS had recurrent painful plaques and fever despite extensive immunosuppressive therapy. Ruxolitinib 20 mg twice daily halted new lesion formation within one month, and treatment was continued without adverse effects [60].

A 66‐year‐old man with JAK2‐positive myelofibrosis developed recurrent SS involving the buttocks, limbs, ears, and fingers. Ruxolitinib 10 mg twice daily induced an 18‐month remission, after which SS recurred in parallel with progression to secondary acute myeloid leukemia (AML) [56].

#### 3.3.2. Ruxolitinib‐Associated SS

Six patients, all with underlying myelofibrosis, developed ruxolitinib‐associated SS. Ruxolitinib was administered for symptom control in five cases and as part of chemotherapy in one. The interval between treatment initiation and SS onset ranged from 3 weeks to 18 months in four patients, while it was not applicable in two cases. Corticosteroid therapy led to rapid resolution of fever and cutaneous lesions in four patients. In one patient, discontinuation of ruxolitinib resulted in remission. In another, dapsone was initiated, but the patient died without symptom resolution because of underlying disease.

A 69‐year‐old man with post‐polycythemia vera myelofibrosis developed pulmonary and cutaneous SS while on ruxolitinib. Biopsy confirmed SS, and systemic corticosteroids led to rapid improvement of both skin lesions and respiratory symptoms [61].

A 77‐year‐old man with transfusion‐dependent myelofibrosis developed painful bullous and erosive lesions along a postoperative wound five weeks after starting ruxolitinib. Biopsies showed ND, and lesions resolved with systemic and topical corticosteroids without discontinuing ruxolitinib [62].

A 66‐year‐old woman with myelofibrosis and recent AML, previously treated with ruxolitinib, presented with a rapidly progressive leg lesion initially suspected to be necrotizing fasciitis. Repeat biopsies confirmed necrotizing SS, and high‐dose prednisone produced marked clinical improvement [63].

A 46‐year‐old man with myelofibrosis receiving ruxolitinib and decitabine developed febrile neutropenia and multiple painful plaques unresponsive to antibiotics. Biopsy confirmed SS, and glucocorticoids resulted in rapid resolution. The temporal pattern suggested multifactorial triggering, including ruxolitinib exposure and hypomethylating therapy [64].

A 59‐year‐old man with postessential thrombocythemia myelofibrosis developed recurrent subcutaneous SS shortly after starting ruxolitinib, with additional flares after drug discontinuation. Each episode responded promptly to systemic corticosteroids, and he later underwent allogeneic stem cell transplantation [65].

A 77‐year‐old woman with postessential thrombocythemia myelofibrosis developed rapidly progressive pustular and ulcerative lesions two weeks after ruxolitinib withdrawal. Biopsy confirmed SS, but she showed minimal response to corticosteroids and dapsone and died shortly because of underlying disease [66].

### 3.4. Quality Assessment

**FIGURE 3:**
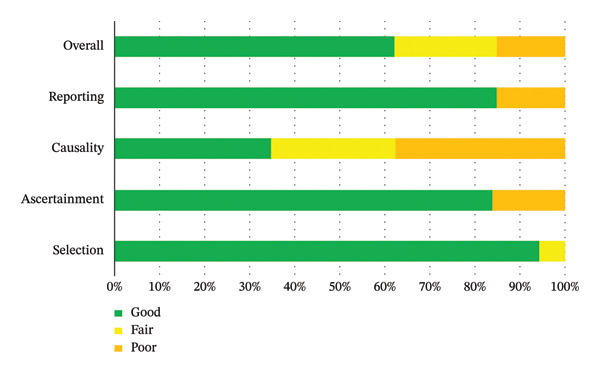
Quality assessment of included case reports and case series across four methodological domains—selection, ascertainment, causality, and reporting—as well as the overall quality rating according to the Murad et al. tool.

The quality assessment demonstrated that most included case reports and case series were rated as good, with consistent strengths in selection, ascertainment, and reporting domains. A smaller proportion were classified as fair or poor, largely due to limitations in causality assessment and incomplete ascertainment. The distribution of ratings across domains is reflected in Figure 3, which shows good‐quality studies comprising the majority. The single observational cohort study was rated good, meeting nearly all methodological criteria aside from participation rate, repeated exposure assessment, blinding, and follow‐up completeness. Overall, the evidence base is acceptable for descriptive synthesis.

## 4. Discussion

PG is a subtype of NDs, which can occur alone or with other inflammatory diseases. Although the pathogenesis is not fully understood, research indicates that abnormal neutrophil chemotaxis, T‐cell‐mediated response, and proinflammatory cytokines play a significant role in disease pathogenesis [67]. In the absence of a standard treatment protocol, several treatment options are available for PG. The choice of treatment depends on various factors, and no single approach is effective for all cases. Some cases are complex and require the use of multiple drugs [3].

Our results indicate that different JAK‐Is are useful for the treatment of PG. Studies show that the JAK/STAT signaling pathway is involved in immune regulation and is implicated in autoimmune and inflammatory diseases [5, 68]. Recent studies showed that the dermis and epidermis of PG patients have overexpression of JAK‐1 and JAK‐3, with lesser expression of JAK‐2 compared to healthy controls [69]. This suggests that targeting these pathways with JAK‐Is could provide a potential therapeutic avenue for PG. PG shows cytokine‐rich inflammation with JAK‐STAT upregulation, whereas SS is more heterogeneous, with IL‐1–driven autoinflammatory forms and malignancy‐associated variants. These differences explain why JAK‐I responsiveness cannot be generalized across all NDs [70]. However, comparable therapeutic benefits have been reported in other neutrophilic or autoinflammatory conditions, such as SAPHO syndrome, VEXAS syndrome, and prurigo nodularis, where JAK‐Is have produced meaningful clinical responses in refractory cases [71–73].

**FIGURE 4:**
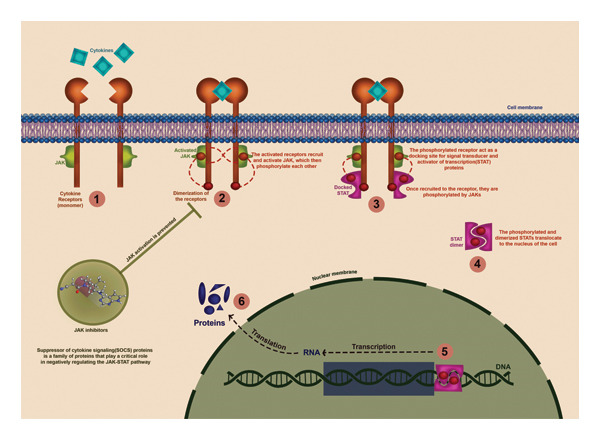
Overview of the JAK‐STAT pathway and mechanism of JAK inhibitors: 1. Cytokine binding to receptors: Extracellular cytokines initiate the signaling cascade when they attach to their corresponding cytokine receptors, which are first found on the cell membrane as inactive monomers. 2. Receptor dimerization and JAK activation: The receptors dimerize in response to cytokine interaction, which draws the corresponding Janus kinases (JAKs) closer together. Because of their close proximity, the JAKs might phosphorylate one another and become activated. 3. Phosphorylation of the receptor and STAT recruitment: Certain tyrosine residues on the cytoplasmic tails of the receptor are phosphorylated by activated JAKs. These phosphorylated residues serve as docking sites for the signal transducers and activators of transcription (STAT) proteins. 4. STAT phosphorylation and dimerization: JAKs phosphorylate STAT proteins once they are recruited. After that, the phosphorylated STATs dimerize, allowing for their translocation to the cell nucleus. 5. Translocation to the nucleus and gene activation: The transcription of target genes involved in immune response, cell development, and differentiation is activated by the STAT dimers when they enter the nucleus and bind to certain DNA sequences. 6. Protein synthesis: These genes are transcriptionally active, producing mRNA, which is then translated into proteins that modulate a variety of biological processes, including immunological responses.

In the literature, five different JAK‐Is have been utilized for the treatment of PG: tofacitinib (inhibiting JAK1, JAK2, and JAK3), baricitinib (inhibiting JAK1 and JAK2), abrocitinib (inhibiting JAK1), upadacitinib (inhibiting JAK1), and ruxolitinib (inhibiting JAK1 and JAK2). Various cytokines and growth factors, such as numerous interleukins, interferons, myeloproliferative leukemia (MPL) viral oncogene, ciliary neurotrophic factor (CNTF), and granulocyte‐macrophage colony‐stimulating factor (GM‐CSF), are influenced by the different signaling pathways of JAK subtypes (Figure 4) [5, 7, 74]. Therefore, a more selective JAK‐I may demonstrate greater efficacy in the treatment of PG.

Although JAK‐Is produced high clinical response rates in PG, recurrence was observed in cases where treatment was stopped. Ulcers reappeared four weeks after baricitinib withdrawal in one patient, and discontinuation of abrocitinib due to an adverse event triggered a flare in another. These findings suggest that JAK‐Is may act primarily as disease‐controlling agents, and abrupt cessation can unmask persistent inflammation. This highlights the potential need for individualized maintenance strategies and careful tapering. Future studies should clarify optimal treatment duration and relapse‐prevention approaches.

Inhibition of the JAK‐STAT Pathway: JAK‐Is: The phosphorylation cascade that results in STAT activation and gene transcription is blocked by JAK‐Is by inhibiting JAK activity, as seen in the figure. Negative Regulation by suppressor of cytokine signaling (SOCS) Proteins: Additionally, the JAK‐STAT system is inherently inhibited by the SOCS proteins, which acts, as feedback control to stop overstimulation.

SS is a type of NDs which can be idiopathic or related to malignancy, inflammatory conditions, or infections. Treatment is based on underlying disease, primarily utilizing corticosteroids and anti‐TNF‐α agents [1]. With a multifactorial pathogenesis, different pathways and factors contribute to the development of SS based on its variant. Our results identified nine cases of SS related to the use of JAK‐Is. Baricitinib, a JAK 1 and 2 inhibitor, showed a favorable response in two cases. Both of which had underlying inflammatory diseases. Baricitinib can influence different types of chemokines like IL‐21, which is important in inflammatory and autoimmune conditions [7, 74]. This inhibition can suppress the underlying inflammatory condition, facilitating the treatment of SS. Ruxolitinib, another JAK1 and JAK2 inhibitor, exhibited variable effects. In a case with SS and underlying MGUS, improvement was noted with no side effect. Conversely, six other cases developed SS after using ruxolitinib for underlying myelofibrosis. By being a JAK1 and JAK2 inhibitors, ruxolitinib can affect many cytokines and growth factors such as different interleukins and interferons as well as GM‐CSF, erythropoietin, the MPL viral oncogene, and CNTF [7].

This disparity in ruxolitinib’s effect on SS may be attributable to the underlying hematologic disease. It is also important to note that approximately 20 percent of SS cases are associated with malignancies, and all six patients in this study had underlying hematologic malignancies [4]. The incidence of SS may be solely due to myelofibrosis or could result from a multifactorial situation. A study discussed SS as a potential adverse reaction to tyrosine kinase inhibitors, which have effects similar to those of JAK‐Is [7, 75]. However, all patients reported in this study had underlying malignancies.

Some studies have suggested that JAK‐Is may potentially provoke cutaneous T‐cell lymphoma in patients with underlying autoimmune disease [76, 77]. However, larger pooled analyses do not support a generalized increase in malignancy risk. A recent meta‐analysis found no higher incidence of malignancy among patients receiving JAK‐Is compared with placebo [78]. In addition, another meta‐analysis evaluating dermatologic indications reported no increased risk of major adverse cardiovascular events associated with JAK‐Is therapy [79]. Meta‐analytic data also indicate that oral JAK‐Is consistently raise LDL and HDL levels, highlighting dyslipidemia as a class‐associated laboratory change requiring routine monitoring [80]. Additionally, a pooled analysis of 26 randomized trials found a higher overall risk of infections, including an increased incidence of herpes zoster in dermatologic populations compared with placebo [81].

Previous reports have described the dual role of biologic therapies, including adalimumab, atezolizumab, and rituximab, in both the induction and treatment of PG [30, 42, 82–84]. Notably, the majority of cases included in our review (62.7%) had received biologic agents prior to initiation of JAK‐Is without achieving clinical improvement.

Also, a recent meta‐analysis by Kaur et al. [85] evaluated systemic therapies for PG, including adalimumab, canakinumab, infliximab, chlorambucil, cyclosporine, cyclophosphamide, and prednisolone. These treatments were associated with a wide range of adverse effects, from mild symptoms such as hypotension and malaise to severe complications including congestive heart failure, septicemia, and death. The overall efficacy of first‐line treatments, including biologics and immunomodulators, was reported at 59%, with a treatment failure rate of 36% and a recurrence rate of 30%.

Given these relatively modest response rates and the potential for serious adverse events, there is a growing rationale for expanding the use of JAK‐Is in PG. They may offer a more effective and potentially safer therapeutic option when used either as monotherapy or in combination with conventional treatments.

This study has several limitations. First, the available evidence is derived from case reports and small case series, which inherently limits the strength of conclusions and increases susceptibility to publication bias, particularly toward positive treatment outcomes. Second, outcome definitions across studies were highly heterogeneous and often nonstandardized, restricting the ability to perform reliable comparative analyses, including monotherapy versus combination therapy. Third, dosing regimens, treatment durations, and concomitant therapies varied widely, further limiting comparability across reports. Fourth, the exclusion of non‐English publications may introduce language bias and could have resulted in the omission of relevant studies. Finally, follow‐up periods were inconsistently reported, preventing meaningful assessment of long‐term efficacy, recurrence rates, and delayed adverse events.

In conclusion, JAK‐Is have been associated with reported clinical improvement in refractory PG without serious short‐term side effects. Based on current evidence, they appear most suitable as adjunct or combination therapies rather than first‐line monotherapy. JAK‐I use is controversial for SS treatment, particularly in patients with underlying malignancies. Given the included evidence consists mainly of case reports and case series, larger studies are needed, and prospective controlled studies are required before routine clinical use.

## Author Contributions

Conceptualization: Mahshid Sadat Ansari and Seyed Mohammad Vahabi; data curation: Saeed Bahramian, Yalda Farahmand, and Seyed Mohammad Vahabi; methodology: Mahshid Sadat Ansari, Nika Kianfar, Sama Heidari, and Farzad Esmaeili; supervision: Mahshid Sadat Ansari; validation: Nika Kianfar, Elnaz Pourgholi, Farzad Esmaeili, and Seyed Mohammad Vahabi; visualization: Huria Memari; Ifa Etesami; writing–original draft preparation: Seyed Mohammad Vahabi, Yalda Farahmand, Farzad Esmaeili, Elnaz Pourgholi, Sama Heidari, and Huria Memari; writing–review and editing: Seyed Mohammad Vahabi Ifa Etesami, Nika Kianfar, Saeed Bahramian, and Mahshid Sadat Ansari.

## Funding

This research received no funding.

## Disclosure

All authors have read and agreed to the published version of the manuscript.

## Conflicts of Interest

The authors declare no conflicts of interest.

## Supporting Information

Additional supporting information can be found online in the Supporting Information section.

## Supporting information


**Supporting Information 1** Supporting file 1 shows the search query by each database and in details.

**Supporting Information 2** Supporting file 2 shows quality assessment of included studies.

**Supporting Information 3** Supporting file 3 shows detailed tables of included studies.

## Data Availability

Data sharing is not applicable to this article, as no new data were created or analyzed in this study.

## References

[1] Weiss E. H. , Ko C. J. , and Leung T. H. , Neutrophilic Dermatoses: A Clinical Update, Current Dermatology Reports. (2022) 11, no. 2, 89–102, 10.1007/s13671-022-00355-8. 35310367 PMC8924564

[2] Palanivel J. , Macbeth A. , and Levell N. , Pyoderma Gangrenosum in Association With Janus Kinase 2 (JAK2V617F) Mutation, Clinical and Experimental Dermatology. (2013) 38, no. 1, 44–46, 10.1111/j.1365-2230.2012.04375.x. 22607468

[3] Maverakis E. , Marzano A. V. , and Le S. T. , Pyoderma Gangrenosum, Nature Reviews Disease Primers. (2020) 6, no. 1, 10.1038/s41572-020-0213-x. 33033263

[4] Orfaly V. E. , Shakshouk H. , Heath M. , Hamilton A. , and Ortega-Loayza A. G. , Sweet syndrome: A Review of Published Cases, Dermatology. (2023) 239, no. 4, 664–669, 10.1159/000530519. 37019090

[5] Xin P. , Xu X. , and Deng C. , The Role of JAK/STAT Signaling Pathway and Its Inhibitors in Diseases, International Immunopharmacology. (2020) 80, 10.1016/j.intimp.2020.106210. 31972425

[6] Crisler W. J. , Eshleman E. M. , and Lenz L. L. , Ligand-Induced IFNGR1 Down-Regulation Calibrates Myeloid Cell IFNγ Responsiveness, Life Science Alliance. (2019) 2, no. 5. 10.26508/lsa.201900447 PMC6778285 31585982

[7] Miot H. A. , Criado P. R. , Castro C. C. Sd , Ianhez M. , Talhari C. , and Ramos P. M. , JAK-STAT Pathway Inhibitors in Dermatology, Anais Brasileiros de Dermatologia. (2023) 98, no. 5, 656–677, 10.1016/j.abd.2023.03.001. 37230920 PMC10404561

[8] Ansari M. S. , Vahabi S. M. , Memari H. , Hosseini F. , Bahramian S. , and Etesami I. , Use of the Oral JAK Inhibitor Tofacitinib in the Treatment of Morphea: A Retrospective Study, Journal of the American Academy of Dermatology. (2025) . 10.1016/j.jaad.2025.05.1377 40374119

[9] Crisler W. J. , Rowley R. , Oke O. et al., Eosinophilic Fasciitis and Morphea Share Gene Signatures of Inflammatory Cell Death, Self-DNA Recognition, and Enhanced JAK/Signal Transducer and Activator of Transcription Signaling, Journal of Investigative Dermatology. (2025) . 10.1016/j.jid.2025.05.023 40451539

[10] Ansari M. S. , Heidari S. , Pourgholi E. , Bahramian S. , Tootoonchi N. , and Vahabi S. M. , Janus Kinase Inhibitors for Treatment of Palmoplantar Pustulosis, Generalized Pustular Psoriasis, and Palmoplantar Pustular Psoriasis: A Systematic Review of the Literature, Health Science Reports. (2026) 9, no. 4, 10.1002/hsr2.72301. PMC13053663 41953903

[11] Page M. J. , McKenzie J. E. , Bossuyt P. M. et al., The PRISMA 2020 Statement: An Updated Guideline for Reporting Systematic Reviews, BMJ. (2021) 372. 10.1136/bmj.n71 PMC8005924 33782057

[12] Murad M. H. , Sultan S. , Haffar S. , and Bazerbachi F. , Methodological Quality and Synthesis of Case Series and Case Reports, BMJ Evidence-Based Medicine. (2018) . 10.1136/bmjebm-2017-110853 PMC6234235 29420178

[13] Lee S. D. , Singla A. , and Harper J. , Safety and Efficacy of Tofacitinib in Combination With Biologic Therapy for Refractory Crohn’s Disease, Inflammatory Bowel Diseases. (2022) 28, no. 2, 309–313, 10.1093/ibd/izab176. 34347103 PMC8804506

[14] Gregory M. H. , Ciorba M. A. , Deepak P. , and Christophi G. P. , Successful Treatment of Pyoderma Gangrenosum With Concomitant Tofacitinib and Infliximab, Inflammatory Bowel Diseases. (2019) 25, no. 7, e87–e88, 10.1093/ibd/izz015. 30753456

[15] Bechard K. and Gniadecki R. , Use of Baricitinib in a Patient With Treatment-Resistant Pyoderma Gangrenosum, SAGE Open Medical Case Reports. (2024) 12, 10.1177/2050313x241235444. PMC10960974 38524384

[16] Yeo T. F. , Labbouz S. , Lawrance N. , Sitaraaman H. B. , Tattersall R. S. , and Cork M. J. , Refractory Pyoderma Gangrenosum in Caucasian Adolescent With Takayasu Arteritis and Life‐Threatening Infections, JEADV Clinical Practice. (2025) 4, no. 1, 234–239, 10.1002/jvc2.534.

[17] Shibuta K. , Hayama K. , Miura K. , and Fujita H. , Successful Treatment of Pyoderma Gangrenosum Complicated by JAK2V617F mutation-Positive Myelofibrosis With Adalimumab and Systemic Steroid, Journal of Cutaneous Immunology and Allergy. (2025) 8, 10.3389/jcia.2025.14079.

[18] Bhowmick K. , Roongta R. , and Dey S. , Refractory Takayasu Arteritis With Recurrent Pyoderma Gangrenosum: A Therapeutic Challenge With Case-Based Review, Clinical Rheumatology. (2023) 42, no. 5, 1469–1477, 10.1007/s10067-023-06506-x. 36637635

[19] Köken Avşar A. , Demirci Yıldırım T. , and Sarı İ. , Tofacitinib Therapy for Severe Pyoderma Gangrenosum in a Patient With Enteropathic Arthritis: A Case-Based Review, Rheumatology International. (2024) 44, no. 10, 2227–2237, 10.1007/s00296-024-05560-1. 38488863

[20] Sathyanarayana V. A. , Roy D. , Nagaraju B. , and Rao V. K. , Tofacitinib in Pyoderma gangrenosum–A Case Series, International Journal of Rheumatic Diseases. (2024) 27, no. 1, 10.1111/1756-185x.14810. 37395471

[21] Bhadresha S. , Connolly A. , Galloway J. , and Walsh S. , A Noduloulcerative Plaque in a Patient With Rheumatoid Arthritis, Clinical and Experimental Dermatology. (2022) 47, no. 1, 226–228, 10.1111/ced.14778. 34263480

[22] Olavarría P. S. , Iturria S. R. , and Castillejo ÓN. , Tofacitinib, a Useful Option for the Treatment of Pyoderma Gangrenosum in an Ulcerative Colitis Patient, Revista Española de Enfermedades Digestivas. (2021) 113, 733–734. 33845581 10.17235/reed.2021.7977/2021

[23] Orfaly V. , Kovalenko I. , Tolkachjov S. , Ortega‐Loayza A. , and Nunley J. , Tofacitinib for the Treatment of Refractory Pyoderma Gangrenosum, Clinical and Experimental Dermatology. (2021) 46, no. 6, 1082–1085, 10.1111/ced.14683. 33864685

[24] Choi A. W. , Abuav R. , Rabizadeh S. M. , Ansari R. , and Marsch A. F. , Recalcitrant and Severe Pyoderma Gangrenosum Attributable to Levamisole-Adulterated Cocaine and Treated Successfully With Oral Tofacitinib, JAAD Case Reports. (2020) 6, no. 9, 939–941, 10.1016/j.jdcr.2020.07.035. 32923571 PMC7475066

[25] Kochar B. , Herfarth N. , Mamie C. , Navarini A. A. , Scharl M. , and Herfarth H. H. , Tofacitinib for the Treatment of Pyoderma Gangrenosum, Clinical Gastroenterology and Hepatology. (2019) 17, no. 5, 991–993, 10.1016/j.cgh.2018.10.047. 30404036

[26] Castro L. G. , JAK Inhibitors: A Novel, Safe, and Efficacious Therapy for Pyoderma Gangrenosum, International Journal of Dermatology. (2023) 62, no. 8, 1088–1093, 10.1111/ijd.16676. 37118975

[27] Narula S. , Chanana K. , Thole A. , Sardana K. , and Muddebihal A. , A Case of Multifocal Pyoderma Gangrenosum With Cyclosporine Induced Neurotoxicity and Its Exquisite Response to Tofacitinib, Indian Dermatology Online Journal. (2025) 16, no. 3, 455–456, 10.4103/idoj.idoj_231_24. 40395592 PMC12088472

[28] Xiao Y. , Liao S. , and Hu D. , Refractory Giant Perianal Pyoderma Gangrenosum Successfully Treated With Tofacitinib, Indian Journal of Dermatology. (2024) 69, no. 4, 343–344, 10.4103/ijd.ijd_1109_23. 39296705 PMC11407559

[29] Sedano R. and Jairath V. , Tofacitinib for the Treatment of Three Immune-Mediated Conditions in One Patient: Ulcerative Colitis, Pyoderma Gangrenosum, and Alopecia Areata, Inflammatory Bowel Diseases. (2021) 27, no. 5, e65–e, 10.1093/ibd/izab005. 33484124

[30] Kim H. S. , Kwon J. E. , and Park Y. J. , Atezolizumab Plus Bevacizumab-Induced Recalcitrant Pyoderma Gangrenosum Treated With Baricitinib: A Case Report, Acta Dermato-Venereologica. (2023) 103, 10.2340/actadv.v103.9646. PMC10413870 37526292

[31] Sitaru S. , Biedermann T. , and Lauffer F. , Successful Treatment of Pyoderma Gangrenosum With Janus Kinase 1/2 Inhibition, JEADV Clinical Practice. (2022) 1, no. 4, 420–423, 10.1002/jvc2.62.

[32] Wang Z. , Li T. , Gong L. , Song Z. , and Piao Y. , Successful Treatment of Multiple Site Involvement Pyoderma Gangrenosum With Baricitinib, International Journal of Dermatology. (2024) 63, no. 10, 1444–1446, 10.1111/ijd.17200. 38727093

[33] Choe S. I. , Shettig A. , and Kody S. , Pyoderma Gangrenosum of the Genitalia, Anus, and Perineum: Two Case Reports and a Review of Published Cases, Sexually Transmitted Diseases. (2024) 51, no. 8, 548–550, 10.1097/olq.0000000000001984. 38647256

[34] Grisé A. , Valere L.-C. , Weinstein D. , and Sami N. , Janus Kinase Inhibitors in the Treatment of Pyoderma Gangrenosum: Case Report and Review, Archives of Dermatological Research. (2024) 316, no. 6, 10.1007/s00403-024-02958-6. 38795155

[35] Ito H. , Noda K. , Saruta M. , and Kurosaka D. , Case Report: Peristomal Pyoderma Gangrenosum Complicated by Rheumatoid Arthritis and Behçet’s Disease Successfully Treated With Baricitinib, International Journal of Rheumatic Diseases. (2024) 27, no. 7, 10.1111/1756-185x.15275. 39046183

[36] Scheinberg M. , Machado L. A. , Castro L. G. M. , Ferreira S. B. , and Michalany N. , Successful Treatment of Ulcerated Pyoderma Gangrenosum With Baricitinib, a Novel JAK Inhibitor, Journal of translational autoimmunity. (2021) 4, 10.1016/j.jtauto.2021.100099. PMC8100614 33997753

[37] Chen P. , Liang J. , and Li C. , Abrocitinib as a Novel Treatment for Multiple Skin Disorders: 3 Case Reports and a Scoping Review, Clinical, Cosmetic and Investigational Dermatology. (2024) 17, 35–40, 10.2147/ccid.s446369. 38204456 PMC10778201

[38] Estrella M. M. E. and Verallo-Rowell V. M. , Pyoderma Gangrenosum Treated With Oral Abrocitinib in a 54-Year-Old Woman: A Case Report, JAAD Case Reports. (2025) 60, 4–6, 10.1016/j.jdcr.2025.02.022. 40353091 PMC12059381

[39] He S.-D. and Tian Y. , Upadacitinib for Ulcerative Colitis and Pyoderma Gangrenosum in a Patient With Schizophrenia on Long-Term Risperidone: A Case Report, World Journal of Gastroenterology. (2025) 31, no. 20, 10.3748/wjg.v31.i20.104038. PMC12146935 40495949

[40] Korytnikova E. , Halasz C. L. , and Sangeorzan E. , Upadacitinib for Pyoderma Gangrenosum: A Case Report and Review of Emerging Evidence, SKIN The Journal of Cutaneous Medicine. (2025) 9, no. 4, 2506–2512, 10.25251/jfe9bk61.

[41] Ramos F. M. , García-Ruíz R. , Vázquez A. A. , and Mercader-García P. , Four-Case Report of Upadacitinib as an Alternative Treatment for Patients With Recalcitrant Pyoderma Gangrenosum, Actas Dermo-Sifiliográficas. (2024) 115, no. 10, 1020–1023, 10.1016/j.ad.2023.05.045. 39032777

[42] Hilton B. , Cleaver D. , and Cleaver L. , 44360 Adalimumab Paradoxically Causing Pyoderma Gangrenosum, Journal of the American Academy of Dermatology. (2023) 89, no. 3, 10.1016/j.jaad.2023.07.058.

[43] Kooybaran N. R. , Korsten P. , Schön M. P. , and Mössner R. , Response of Rheumatoid Arthritis‐Associated Pyoderma Gangrenous to the JAK1 Inhibitor Upadacitinib, 2022. 10.1111/ddg.14716 35267240

[44] Park S. , St Pierre J. , Onajin O. , and Rubin D. T. , Successful Treatment of Severe Pyoderma Gangrenosum and Ulcerative Colitis With Upadacitinib, ACG Case Reports Journal. (2024) 11, no. 10, 10.14309/crj.0000000000001531. PMC11469854 39399247

[45] Tanida S. , Kubo R. , and Yoshii S. , Upadacitinib Plus Intensive Granulocyte and Monocyte Adsorptive Apheresis for Ulcerative Colitis Achieved Ulcer Healing for Pyoderma Gangrenosum, Journal of Clinical Medicine Research. (2023) 15, no. 10-11, 446–455, 10.14740/jocmr5005. 38189038 PMC10769604

[46] Van Eycken L. , Dens A.-C. , de Vlam K. , Neerinckx B. , and De Haes P. , Resolution of Therapy-Resistant Pyoderma Gangrenosum With Upadacitinib, JAAD Case Reports. (2023) 37, 89–91, 10.1016/j.jdcr.2023.05.016. 37342401 PMC10277287

[47] Dos Santos M. R. , Ianhez M. , Ribeiro B. N. , de Queiroz B. B. , and Miot H. A. , Refractory Pyoderma Gangrenosum Associated With Rheumatoid Arthritis Successfully Treated With Upadacitinib. Comments on: JAK Inhibitors: A Novel, Safe, and Efficacious Therapy for Pyoderma Gangrenosum, International Journal of Dermatology. (2023) 62, no. 11, 10.1111/ijd.16791. 37424391

[48] Jimenez P. M. P. , Tabib S. , Abbott B. , and Melmed G. , Successful Outcome Treating Pyoderma Gangrenosum and Pouchitis With Upadacitinib, ACG Case Reports Journal. (2024) 11, no. 8, 10.14309/crj.0000000000001442. PMC11319309 39139848

[49] Mao X.-Y. , Yang Y.-Y. , and Tian L. , A Win-Win Solution: Remarkable Reversal of Foot Necrosis in Ulcerative Colitis, Gastroenterology. (2025) 169, no. 1, 30–33, 10.1053/j.gastro.2024.11.021. 39653256

[50] Mendolaro M. , Morello E. , Salacone P. , and Rocca R. , A Case of Refractory Severe Pyoderma Gangrenosum Successfully Treated With Upadacitinib, Digestive and Liver Disease. (2024) 56, no. 7, 10.1016/j.dld.2024.03.016. 38644101

[51] Patel M. , Woo P.-n , and Ahmed F. , BI24 A Case of Refractory Pyoderma Gangrenosum Successfully Treated With Upadacitinib, British Journal of Dermatology. (2025) 193, no. Supplement_1, ljaf085–ljaf433, 10.1093/bjd/ljaf085.433.

[52] Taha M. R. , Nguyen H. P. , and Tyring S. K. , Upadacitinib Monotherapy for Treatment of Pyoderma Gangrenosum, American Journal of Medicine Open. (2025) 14, 10.1016/j.ajmo.2025.100112. PMC12365188 40843451

[53] Zaher A. and Castillo M. , S3860 Successful Treatment of Refractory Peristomal Pyoderma Gangrenosum in an Ulcerative Colitis Patient With Upadacitinib: A Case Report, Official journal of the American College of Gastroenterology| ACG.(2024) 119, no. 10S, S2521–S2522, 10.14309/01.ajg.0001044808.71041.dd.

[54] Nasifoglu S. , Heinrich B. , and Welzel J. , Successful Therapy for Pyoderma Gangrenosum With a Janus Kinase 2 Inhibitor, British Journal of Dermatology. (2018) 179, no. 2, 504–505, 10.1111/bjd.16468. 29451690

[55] Shanmugam V. K. , McNish S. , and Shara N. , Chronic Leg Ulceration Associated With Polycythemia Vera Responding to Ruxolitinib (Jakafi), Journal of Foot and Ankle Surgery. (2013) 52, no. 6, 781–785, 10.1053/j.jfas.2013.07.003. PMC3925681 23953278

[56] Melboucy-Belkhir S. , Brigant F. , Khentache R. , Bouketouche M. , Garidi R. , and Brihaye B. , Sweet syndrome Successfully Treated With Ruxolitinib in JAK-2 Positive Myeloproliferative Disorder, Int Arch Intern Med. (2017) 2, 8–10.

[57] Colina M. , Barisani A. , Gualandi A. , Poli F. , and Campana G. , Successful Use of Filgotinib in the Treatment of Refractory Rheumatoid Arthritis-Associated Sweet syndrome, Journal ISSN. (2025) 2766.

[58] Nousari Y. , Wu B. , and Valenzuela G. , Successful Use of Baricitinib in the Treatment of Refractory Rheumatoid Arthritis‐Associated Sweet Syndrome, Clinical and Experimental Dermatology. (2021) 46, no. 7, 1330–1332, 10.1111/ced.14712. 33914946

[59] Zhang H. , Xia P. , Wu N. , Chen J. , and Liu Y. , Neutrophilic Dermatosis of the Dorsal Hands Treated With Baricitinib, Clinical and Experimental Dermatology. (2023) 48, no. 11, 1274–1276, 10.1093/ced/llad245. 37477416

[60] Korbl J. , Smith A. , and Wood B. , Refractory Sweet Syndrome Successfully Treated With Ruxolitinib, Australasian Journal of Dermatology. (2022) Wiley.

[61] Chou C. and Chatterjee A. B. , Pulmonary Sweet Syndrome in a Patient With Myelofibrosis, Chest. (2023) 164, no. 4, A5545–A5546, 10.1016/j.chest.2023.07.3586.

[62] Jiang M. , Tran A. K. , and Marshman G. , A Neutrophilic Dermatosis Following Treatment of Myelofibrosis With Ruxolitinib: An Emerging Phenomenon?, Australasian Journal of Dermatology. (2021) 62, no. 4, 10.1111/ajd.13717. 34529266

[63] Gowda A. , Christensen L. , Polly S. , and Barlev D. , Necrotizing Neutrophilic Dermatosis: A Diagnostic Challenge With a Need for Multi-Disciplinary Recognition, a Case Report, Annals of Medicine and Surgery. (2020) 57, 299–302, 10.1016/j.amsu.2020.07.037. 32874559 PMC7452005

[64] Thebo U. , Tummala S. , Nassereddine S. , and Haroun F. , An Atypical Presentation of Sweet’s Syndrome in a Myelofibrosis Patient, BMJ Case Reports CP. (2019) 12, no. 3, 10.1136/bcr-2018-228076. PMC6424381 30852515

[65] Sakoda T. , Kanamitsu Y. , and Mori Y. , Recurrent Subcutaneous Sweet’s Disease in a Myelofibrosis Patient Treated With Ruxolitinib Before Allogeneic Stem Cell Transplantation, Internal Medicine. (2017) 56, no. 18, 2481–2485, 10.2169/internalmedicine.8491-16. 28824063 PMC5643178

[66] Chatterjee B. , Rqieh U. , Greaves P. , Piras D. , Firth J. , and Saja K. , Sweet syndrome as Terminal Event in Ruxolitinib-Treated Myelofibrosis, British Journal of Haematology. (2015) 169, no. 3, 10.1111/bjh.13334. 25707420

[67] Caproni M. , Antiga E. , and Volpi W. , The Treg/Th17 Cell Ratio is Reduced in the Skin Lesions of Patients With Pyoderma Gangrenosum, British Journal of Dermatology. (2015) 173, no. 1, 275–278, 10.1111/bjd.13670. 25601218

[68] Xue C. , Yao Q. , and Gu X. , Evolving Cognition of the JAK-STAT Signaling Pathway: Autoimmune Disorders and Cancer, Signal Transduction and Targeted Therapy. (2023) 8, no. 1, 10.1038/s41392-023-01468-7. PMC10196327 37208335

[69] Alves de Medeiros A. K. , Speeckaert R. , Desmet E. , Van Gele M. , De Schepper S. , and Lambert J. , JAK3 as an Emerging Target for Topical Treatment of Inflammatory Skin Diseases, PLoS One. (2016) 11, no. 10, 10.1371/journal.pone.0164080. PMC5053514 27711196

[70] Cohen P. R. , Sweet’s Syndrome–A Comprehensive Review of an Acute Febrile Neutrophilic Dermatosis, Orphanet Journal of Rare Diseases. (2007) 2, no. 1, 10.1186/1750-1172-2-34. PMC1963326 17655751

[71] Fazeli P. , Bahramian S. , and Farahmand K. , JAK Inhibitors for Treatment of SAPHO Syndrome: A Systematic Review of 72 Cases, ACR Open Rheumatology. (2025) 7, no. 10, 10.1002/acr2.70101. PMC12515502 41077060

[72] Bahramian S. , Fazeli P. , and Rafati A. , JAK Inhibitors for Treatment of VEXAS Syndrome: A Systematic Review of 186 Cases, Dermatology Research and Practice. (2025) 2025, no. 1, 10.1155/drp/9127126. PMC12449113 40977752

[73] Vahabi S. M. , Heidari S. , Pourgholi E. et al., Janus Kinase Inhibitors for the Treatment of Prurigo Nodularis: A Systematic Review of 211 Patients, Journal of Dermatology. (2026) . 10.1111/1346-8138.70150 41645656

[74] Mesas‐Fernández A. , Bodner E. , Hilke F. J. , Meier K. , Ghoreschi K. , and Solimani F. , Interleukin‐21 in Autoimmune and Inflammatory Skin Diseases, European Journal of Immunology. (2023) 53, no. 4, 10.1002/eji.202250075. 36811452

[75] Yang J. J. , Maloney N. J. , Nguyen K. A. , Worswick S. , Smogorzewski J. , and Bach D. Q. , Sweet Syndrome as an Adverse Reaction to Tyrosine Kinase Inhibitors: A Review, Dermatologic Therapy. (2021) 34, no. 1, 10.1111/dth.14461. 33112465

[76] Vahabi S. M. , Bahramian S. , and Esmaeili F. , JAK Inhibitors in Cutaneous T-Cell Lymphoma: Friend or Foe? A Systematic Review of the Published Literature, Cancers. (2024) 16, no. 5, 10.3390/cancers16050861. PMC10930951 38473222

[77] Etesami I. , Ansari M. S. , and Pourgholi E. , Drug‐and Vaccine‐Induced Cutaneous T‐Cell Lymphoma: A Systematic Review of the Literature, Journal of skin cancer. (2025) 2025, no. 1, 10.1155/jskc/3103865. PMC11986929 40226161

[78] Russell M. D. , Stovin C. , and Alveyn E. , JAK Inhibitors and the Risk of Malignancy: A Meta-Analysis Across Disease Indications, Annals of the Rheumatic Diseases. (2023) 82, no. 8, 1059–1067, 10.1136/ard-2023-224049. 37247942 PMC10359573

[79] Ingrassia J. P. , Maqsood M. H. , and Gelfand J. M. , Cardiovascular and Venous Thromboembolic Risk With JAK Inhibitors in Immune-Mediated Inflammatory Skin Diseases: A Systematic Review and Meta-Analysis, JAMA dermatology. (2024) 160, no. 1, 28–36, 10.1001/jamadermatol.2023.4090. 37910098 PMC10620674

[80] Isufi D. , Javanmardi N. , and Jensen M. B. , Risk of Dyslipidemia Associated With Oral Janus Kinase Inhibitors: A Systematic Review and Meta‐Analysis of Randomized Placebo‐Controlled Trials, International Journal of Dermatology. (2026) 65, no. 4, 737–745, 10.1111/ijd.70122. 41160040

[81] Isufi D. , Jensen M. B. , Loft N. , Skov L. , Elberling J. , and Alinaghi F. , Risk of Infections During Treatment With Oral Janus Kinase Inhibitors in Randomized Placebo-Controlled Trials: A Systematic Review and Meta-Analysis, JAAD international. (2025) 18, 106–116, 10.1016/j.jdin.2024.09.012. 39717054 PMC11664075

[82] Georgakopoulos J. R. , Rohekar G. , and Lovegrove F. E. , A Case of Rituximab-Induced Pyoderma Gangrenosum, JAAD Case Reports. (2018) 4, no. 10, 979–981, 10.1016/j.jdcr.2018.09.003. 30406172 PMC6214884

[83] Klumpp A. , Luessi F. , and Engel S. , Ocrelizumab-Induced Vulvovaginal Pyoderma Gangrenosum in a Patient With Relapsing-Remitting Multiple Sclerosis, JAAD Case Reports. (2022) 28, 24–27, 10.1016/j.jdcr.2022.08.005. 36097621 PMC9463556

[84] Kohandel K. , Ala S. , Tamizifar B. , Karaminia M. , and Sahraian M. , Pyoderma Gangrenosum in a Patient With Multiple Sclerosis Under Natalizumab Treatment: A Case Report, BMC Neurology. (2025) 25, no. 1, 10.1186/s12883-025-04146-z. PMC11963391 40175973

[85] Kaur M. , Diaz M. J. , and Anthony M. , Treatments for Pyoderma Gangrenosum: A Systematic Review and Single‐Arm Meta‐Analysis of Systemic Therapies, International Wound Journal. (2025) 22, no. 8, 10.1111/iwj.70733. PMC12311392 40740034

