# Supplementary material for: JAK Inhibitors for Treatment of Pyoderma Gangrenosum and Sweet Syndrome: A Systematic Review of Published Case Reports
Source: Dermatol Res Pract. 2026 Jul 18;2026:7086209. doi: 10.1155/drp/7086209 (PMC13379896; doi:10.1155/drp/7086209)
Supplement: Supplementary file 1 — Supporting Information 1 Supporting file 1 shows the search query by each database and in details. [file DRP-2026-7086209-s002.docx]

**Search Date: 7-25-2025**

**PubMed/MEDLINE:**

1. “Sweet syndrome”[mh] OR “Pyoderma Gangrenosum“[mh] OR “Pyoderma“[mh] OR Sweet syndrome[tiab] OR Syndrome, Sweet[tiab] OR Dermatosis, Neutrophilic, Febrile, Acute[tiab] OR Acute Febrile Neutrophilic Dermatosis[tiab] OR Sweet's Syndrome[tiab] OR Sweets Syndrome[tiab] OR Syndrome, Sweet's[tiab] OR Gomm Button Disease[tiab] OR Disease, Gomm Button[tiab] OR Gomm-Button Disease[tiab] OR Disease, Gomm-Button[tiab] OR Neutrophilic Dermatosis, Acute Febrile[tiab] OR acute febrile neutrophil dermatosis[tiab] OR acute febrile neutrophilic dermatitis[tiab] OR dermatosis, acute febrile neutrophilic[tiab] OR Gomm-Button disease[tiab] OR neutrophilic dermatosis, acute febrile[tiab] OR dermatitis ulcerosa[tiab] OR ulcerative dermatitis[tiab] OR pyodermatitis[tiab] OR pyodermia[tiab] OR pyodermitis[tiab] OR pyroderma[tiab] OR pyoderma[tiab]

2. “Janus Kinase Inhibitors”[mh] OR Janus Kinase Inhibitors[tiab] OR Inhibitors, Janus Kinase[tiab] OR Kinase Inhibitors, Janus[tiab] OR JAK Inhibitors[tiab] OR Inhibitors, JAK[tiab] OR Janus Kinase Inhibitor[tiab] OR Inhibitor, Janus Kinase[tiab] OR Kinase Inhibitor, Janus[tiab] OR JAK Inhibitor[tiab] OR Inhibitor, JAK[tiab] OR JAK inhibitor[tiab] OR Janus kinase inhibitors[tiab] OR Janus tyrosine kinase inhibitor[tiab] OR Janus kinase inhibitor[tiab] OR cibinqo[tiab] OR pf 04965842[tiab] OR pf04965842[tiab] OR abrocitinib[tiab] OR incb 028050[tiab] OR incb 28050[tiab] OR incb028050[tiab] OR ly 3009104[tiab] OR ly3009104[tiab] OR olumiant[tiab] OR baricitinib[tiab] OR jte 052[tiab] OR jte 052a[tiab] OR jte052[tiab] OR leo 124249[tiab] OR leo124249[tiab] OR delgocitinib[tiab] OR fedratinib dihydrochloride[tiab] OR fedratinib dihydrochloride monohydrate[tiab] OR fedratinib hydrochloride[tiab] OR inrebic[tiab] OR sar302503[tiab] OR tg 101348[tiab] OR tg101348[tiab] OR fedratinib[tiab] OR filgotinib 2 butenedioate[tiab] OR filgotinib hydrochloride[tiab] OR filgotinib maleate[tiab] OR glpg 0634[tiab] OR glpg0634[tiab] OR gs 6034[tiab] OR gs6034[tiab] OR jyseleca[tiab] OR apoquel[tiab] OR oclacitinib maleate[tiab] OR oclacitinib[tiab] OR onx 0803[tiab] OR pacritinib citrate[tiab] OR pacritinib hydrochloride[tiab] OR sb 1518[tiab] OR sb1518[tiab] OR vonjo[tiab] OR pacritinib[tiab] OR asp015k[tiab] OR peficitinib hydrobromide[tiab] OR peficitinib[tiab] OR incb 18424[tiab] OR incb 424[tiab] OR incb018424[tiab] OR incb18424[tiab] OR jakafi[tiab] OR jakavi[tiab] OR kks 278[tiab] OR opzelura[tiab] OR ruxolitinib maleate[tiab] OR ruxolitinib phosphate[tiab] OR ruxolitinib[tiab] OR cgb 500[tiab] OR cgb500[tiab] OR cp 690 550[tiab] OR cp 690, 550[tiab] OR cp 690550[tiab] OR cp 690550 10[tiab] OR cp690 550[tiab] OR cp690, 550[tiab] OR cp690550[tiab] OR cp690550 10[tiab] OR pgn 600[tiab] OR tasocitinib[tiab] OR tasocitinib citrate[tiab] OR tofacitinib citrate[tiab] OR xeljanz[tiab] OR xeljanz xr[tiab] OR tofacitinib[tiab] OR abt 494[tiab] OR abt494[tiab] OR rinvoq[tiab] OR upadacitinib 2, 3 dihydroxybutanedioate[tiab] OR upadacitinib hemihydrate[tiab] OR upadacitinib hydrate[tiab] OR upadacitinib tartrate[tiab] OR upadacitinib[tiab]

3. #1 AND #2

**Embase**

1. ‘acute febrile neutrophilic dermatosis’/exp OR ‘pyoderma gangrenosum’/exp OR ‘pyoderma’/exp OR ‘Sweet syndrome’:ab,ti OR ‘Syndrome, Sweet’:ab,ti OR ‘Dermatosis, Neutrophilic, Febrile, Acute’:ab,ti OR ‘Acute Febrile Neutrophilic Dermatosis’:ab,ti OR ‘Sweets Syndrome’:ab,ti OR ‘Gomm Button Disease’:ab,ti OR ‘Disease, Gomm Button’:ab,ti OR ‘Gomm-Button Disease’:ab,ti OR ‘Disease, Gomm-Button’:ab,ti OR ‘Neutrophilic Dermatosis, Acute Febrile’:ab,ti OR ‘acute febrile neutrophil dermatosis’:ab,ti OR ‘acute febrile neutrophilic dermatitis’:ab,ti OR ‘dermatosis, acute febrile neutrophilic’:ab,ti OR ‘Gomm-Button disease’:ab,ti OR ‘neutrophilic dermatosis, acute febrile’:ab,ti OR ‘dermatitis ulcerosa’:ab,ti OR ‘ulcerative dermatitis’:ab,ti OR ‘pyodermatitis’:ab,ti OR ‘pyodermia’:ab,ti OR ‘pyodermitis’:ab,ti OR ‘pyroderma’:ab,ti OR ‘pyoderma’:ab,ti

2. ‘Janus kinase inhibitor’/exp OR ‘Abrocitinib’/exp OR ‘Baricitinib’/exp OR ‘Delgocitinib’/exp OR ‘Fedratinib’/exp OR ‘Filgotinib’/exp OR ‘Oclacitinib’/exp OR ‘Pacritinib’/exp OR ‘Peficitinib’/exp OR ‘Ruxolitinib’/exp OR ‘Tofacitinib’/exp OR ‘Upadacitinib’/exp OR ‘Janus Kinase Inhibitors’:ab,ti OR ‘Inhibitors, Janus Kinase’:ab,ti OR ‘Kinase Inhibitors, Janus’:ab,ti OR ‘JAK Inhibitors’:ab,ti OR ‘Inhibitors, JAK’:ab,ti OR ‘Janus Kinase Inhibitor’:ab,ti OR ‘Inhibitor, Janus Kinase’:ab,ti OR ‘Kinase Inhibitor, Janus’:ab,ti OR ‘JAK Inhibitor’:ab,ti OR ‘Inhibitor, JAK’:ab,ti OR ‘JAK inhibitor’:ab,ti OR ‘Janus kinase inhibitors’:ab,ti OR ‘Janus tyrosine kinase inhibitor’:ab,ti OR ‘Janus kinase inhibitor’:ab,ti OR ‘cibinqo’:ab,ti OR ‘n [3 (methyl 7 hydropyrrolo [2, 3 d] pyrimidin 4 ylamino) cyclobutyl] 1 propanesulfonamide’:ab,ti OR ‘n [3 (methyl 7 hydropyrrolo [2, 3 d] pyrimidin 4 ylamino) cyclobutyl] propane 1 sulfonamide’:ab,ti OR ‘n [3 (methyl 7h pyrrolo [2, 3 d] pyrimidin 4 ylamino) cyclobutyl] 1 propanesulfonamide’:ab,ti OR ‘n [3 (methyl 7h pyrrolo [2, 3 d] pyrimidin 4 ylamino) cyclobutyl] propane 1 sulfonamide’:ab,ti OR ‘n [3 (methylpyrrolo [2, 3 d] pyrimidin 4 ylamino) cyclobutyl] 1 propanesulfonamide’:ab,ti OR ‘n [3 (methylpyrrolo [2, 3 d] pyrimidin 4 ylamino) cyclobutyl] propane 1 sulfonamide’:ab,ti OR ‘n [3 [methyl (7 hydropyrrolo [2, 3 d] pyrimidin 4 yl) amino] cyclobutyl] 1 propanesulfonamide’:ab,ti OR ‘n [3 [methyl (7 hydropyrrolo [2, 3 d] pyrimidin 4 yl) amino] cyclobutyl] propane 1 sulfonamide’:ab,ti OR ‘n [3 [methyl (7h pyrrolo [2, 3 d] pyrimidin 4 yl) amino] cyclobutyl] 1 propanesulfonamide’:ab,ti OR ‘n [3 [methyl (7h pyrrolo [2, 3 d] pyrimidin 4 yl) amino] cyclobutyl] propane 1 sulfonamide’:ab,ti OR ‘pf 04965842’:ab,ti OR ‘pf 4965842’:ab,ti OR ‘pf04965842’:ab,ti OR ‘pf4965842’:ab,ti OR ‘abrocitinib’:ab,ti OR ‘1 (ethylsulfonyl) 3 [4 (7h pyrrolo [2, 3 d] pyrimidin 4 yl) 1h 1 pyrazolyl] 3 azetidineacetonitrile’:ab,ti OR ‘1 (ethylsulfonyl) 3 [4 (7h pyrrolo [2, 3 d] pyrimidin 4 yl) 1h pyrazol 1 yl] 3 azetidineacetonitrile’:ab,ti OR ‘2 (3 (4 (3h pyrrolo [2, 3 d] pyrimidin 4 yl) 1h 1 pyrazolyl) 1 (ethylsulfonyl) 3 azetidinyl) acetonitrile’:ab,ti OR ‘2 (3 (4 (3h pyrrolo [2, 3 d] pyrimidin 4 yl) 1h 1 pyrazolyl) 1 (ethylsulfonyl) 3 azetidyl) acetonitrile’:ab,ti OR ‘2 (3 (4 (3h pyrrolo [2, 3 d] pyrimidin 4 yl) 1h pyrazol 1 yl) 1 (ethylsulfonyl) azetidin 3 yl) acetonitrile’:ab,ti OR ‘[1 (ethanesulfonyl) 3 [4 (7 hydropyrrolo [2, 3 d] pyrimidin 4 yl) 1 hydropyrazol 1 yl] azetidin 3 yl] ethanenitrile’:ab,ti OR ‘[1 (ethanesulfonyl) 3 [4 (7h pyrrolo [2, 3 d] pyrimidin 4 yl) 1h 1 pyrazolyl] 3 azetidinyl] ethanenitrile’:ab,ti OR ‘[1 (ethanesulfonyl) 3 [4 (7h pyrrolo [2, 3 d] pyrimidin 4 yl) 1h 1 pyrazolyl] 3 azetidyl] ethanenitrile’:ab,ti OR ‘[1 (ethanesulfonyl) 3 [4 (7h pyrrolo [2, 3 d] pyrimidin 4 yl) 1h pyrazol 1 yl] azetidin 3 yl] ethanenitrile’:ab,ti OR ‘[1 (ethylsulfonyl) 3 [4 (1h pyrrolo [2, 3 d] pyrimidin 4 yl) 1h 1 pyrazolyl] 3 azetidinyl] acetonitrile’:ab,ti OR ‘[1 (ethylsulfonyl) 3 [4 (1h pyrrolo [2, 3 d] pyrimidin 4 yl) 1h 1 pyrazolyl] 3 azetidyl] acetonitrile’:ab,ti OR ‘[1 (ethylsulfonyl) 3 [4 (1h pyrrolo [2, 3 d] pyrimidin 4 yl) 1h pyrazol 1 yl] 3 azetidinyl] acetonitrile’:ab,ti OR ‘[1 (ethylsulfonyl) 3 [4 (7h pyrrolo [2, 3 d] pyrimidin 4 yl) 1h 1 pyrazolyl] 3 azetidinyl] ethanenitrile’:ab,ti OR ‘[1 (ethylsulfonyl) 3 [4 (7h pyrrolo [2, 3 d] pyrimidin 4 yl) 1h 1 pyrazolyl] 3 azetidyl] ethanenitrile’:ab,ti OR ‘[1 (ethylsulfonyl) 3 [4 (7h pyrrolo [2, 3 d] pyrimidin 4 yl) 1h pyrazol 1 yl] azetidin 3 yl] ethanenitrile’:ab,ti OR ‘incb 028050’:ab,ti OR ‘incb 28050’:ab,ti OR ‘incb028050’:ab,ti OR ‘incb28050’:ab,ti OR ‘ly 3009104’:ab,ti OR ‘ly3009104’:ab,ti OR ‘olumiant’:ab,ti OR ‘baricitinib’:ab,ti OR ‘3 [3 methyl 6 (7h pyrrolo [2, 3 d] pyrimidin 4 yl) 1, 6 diazaspiro [3.4] octan 1 yl] 3 oxopropanenitrile’:ab,ti OR ‘jte 052’:ab,ti OR ‘jte 052a’:ab,ti OR ‘jte052’:ab,ti OR ‘jte052a’:ab,ti OR ‘leo 124249’:ab,ti OR ‘leo 124249a’:ab,ti OR ‘leo124249’:ab,ti OR ‘leo124249a’:ab,ti OR ‘delgocitinib’:ab,ti OR ‘fedratinib dihydrochloride’:ab,ti OR ‘fedratinib dihydrochloride monohydrate’:ab,ti OR ‘fedratinib hydrochloride’:ab,ti OR ‘inrebic’:ab,ti OR ‘n (1, 1 dimethylethyl) 3 [5 methyl 2 [4 (2 pyrrolidin 1 ylethoxy) phenylamino] pyrimidin 4 ylamino] benzenesulfonamide’:ab,ti OR ‘n (1, 1 dimethylethyl) 3 [5 methyl 2 [4 [2 (1 pyrrolidinyl) ethoxy] phenylamino] 4 pyrimidinylamino] benzenesulfonamide’:ab,ti OR ‘n (1, 1 dimethylethyl) 3 [5 methyl 2 [4 [2 (1 pyrrolidyl) ethoxy] phenylamino] 4 pyrimidylamino] benzenesulfonamide’:ab,ti OR ‘n (1, 1 dimethylethyl) 3 [ [5 methyl 2 [4 [2 (pyrrolidin 1 yl) ethoxy] anilino] pyrimidin 4 yl] amino] benzenesulfonamide’:ab,ti OR ‘n (1, 1 dimethylethyl) 3 [ [5 methyl 2 [ [4 (2 pyrrolidin 1 ylethoxy) phenyl] amino] pyrimidin 4 yl] amino] benzenesulfonamide’:ab,ti OR ‘n (1, 1 dimethylethyl) 3 [ [5 methyl 2 [ [4 [2 (1 pyrrolidinyl) ethoxy] phenyl] amino] 4 pyrimidinyl] amino] benzenesulfonamide’:ab,ti OR ‘n (1, 1 dimethylethyl) 3 [ [5 methyl 2 [ [4 [2 (1 pyrrolidyl) ethoxy] phenyl] amino] 4 pyrimidyl] amino] benzenesulfonamide’:ab,ti OR ‘n (1, 1 dimethylethyl) 3 [ [5 methyl 2 [ [4 [2 (pyrrolidin 1 yl) ethoxy] phenyl] amino] pyrimidin 4 yl] amino] benzenesulfonamide’:ab,ti OR ‘n tert butyl 3 [5 methyl 2 [4 (2 pyrrolidin 1 ylethoxy) phenylamino] pyrimidin 4 ylamino] benzenesulfonamide’:ab,ti OR ‘n tert butyl 3 [5 methyl 2 [4 [2 (1 pyrrolidinyl) ethoxy] phenylamino] 4 pyrimidinylamino] benzenesulfonamide’:ab,ti OR ‘n tert butyl 3 [5 methyl 2 [4 [2 (1 pyrrolidyl) ethoxy] phenylamino] 4 pyrimidylamino] benzenesulfonamide’:ab,ti OR ‘n tert butyl 3 [ [5 methyl 2 [4 [2 (1 pyrrolidinyl) ethoxy] anilino] 4 pyrimidinyl] amino] benzenesulfonamide’:ab,ti OR ‘n tert butyl 3 [ [5 methyl 2 [4 [2 (1 pyrrolidyl) ethoxy] anilino] 4 pyrimidyl] amino] benzenesulfonamide’:ab,ti OR ‘n tert butyl 3 [ [5 methyl 2 [4 [2 (pyrrolidin 1 yl) ethoxy] anilino] pyrimidin 4 yl] amino] benzenesulfonamide’:ab,ti OR ‘n tert butyl 3 [ [5 methyl 2 [ [4 (2 pyrrolidin 1 ylethoxy) phenyl] amino] pyrimidin 4 yl] amino] benzenesulfonamide’:ab,ti OR ‘sar 302503’:ab,ti OR ‘sar 302503a’:ab,ti OR ‘sar302503’:ab,ti OR ‘sar302503a’:ab,ti OR ‘tg 101348’:ab,ti OR ‘tg101348’:ab,ti OR ‘fedratinib’:ab,ti OR ‘filgotinib 2 butenedioate’:ab,ti OR ‘filgotinib hydrochloride’:ab,ti OR ‘filgotinib maleate’:ab,ti OR ‘g 146034’:ab,ti OR ‘g 146034 101’:ab,ti OR ‘g 146034-101’:ab,ti OR ‘g146034’:ab,ti OR ‘g146034 101’:ab,ti OR ‘g146034-101’:ab,ti OR ‘glpg 0634’:ab,ti OR ‘glpg0634’:ab,ti OR ‘gs 6034’:ab,ti OR ‘gs6034’:ab,ti OR ‘jyseleca’:ab,ti OR ‘n [5 [4 (1, 1 dioxothiomorpholinomethyl) phenyl] 1, 2, 4 triazolo [1, 5 a] pyridin 2 yl] cyclopropanecarboxamide’:ab,ti OR ‘n [5 [4 (1, 1 dioxothiomorpholinomethyl) phenyl] 1, 2, 4 triazolo [1, 5 a] pyridin 2 yl] cyclopropanecarboxamide 2 butenedioate’:ab,ti OR ‘n [5 [4 (1, 1 dioxothiomorpholinomethyl) phenyl] 1, 2, 4 triazolo [1, 5 a] pyridin 2 yl] cyclopropanecarboxamide but 2 enedioate’:ab,ti OR ‘n [5 [4 [ (1, 1 dioxido 4 thiomorpholinyl) methyl] phenyl] 1, 2, 4 triazolo [1, 5 a] pyridin 2 yl] cyclopropanecarboxamide’:ab,ti OR ‘n [5 [4 [ (1, 1 dioxido 4 thiomorpholinyl) methyl] phenyl] 1, 2, 4 triazolo [1, 5 a] pyridin 2 yl] cyclopropanecarboxamide 2 butenedioate’:ab,ti OR ‘n [5 [4 [ (1, 1 dioxidothiomorpholin 4 yl) methyl] phenyl] [1, 2, 4] triazolo [1, 5 a] pyridin 2 yl] cyclopropanecarboxamide’:ab,ti OR ‘n [5 [4 [ (1, 1 dioxo 1, 4 thiazinan 4 yl) methyl] phenyl] [1, 2, 4] triazolo [1, 5 a] pyridin 2 yl] cyclopropanecarboxamide’:ab,ti OR ‘n [5 [4 [ (1, 1 dioxothiomorpholin 4 yl) methyl] phenyl] 1, 2, 4 triazolo [1, 5 a] pyridin 2 yl] cyclopropanecarboxamide’:ab,ti OR ‘n [5 [4 [ (1, 1 dioxothiomorpholin 4 yl) methyl] phenyl] 1, 2, 4 triazolo [1, 5 a] pyridin 2 yl] cyclopropanecarboxamide but 2 enedioate’:ab,ti OR ‘n [5 [4 [ (1, 1 dioxothiomorpholin 4 yl) methyl] phenyl] [1, 2, 4] triazolo [1, 5 a] pyridin 2 yl] cyclopropanecarboxamide’:ab,ti OR ‘n [5 [4 [ (1, 1 dioxothiomorpholin 4 yl) methyl] phenyl] [1, 2, 4] triazolo [1, 5 a] pyridin 2 yl] cyclopropanecarboxamide but 2 enedioate’:ab,ti OR ‘filgotinib’:ab,ti OR ‘apoquel’:ab,ti OR ‘n methyl 1 [4 [methyl (7 hydropyrrolo [2, 3 d] pyrimidin 4 yl) amino] cyclohexyl] methanesulfonamide’:ab,ti OR ‘n methyl 1 [4 [methyl (7h pyrrolo [2, 3 d] pyrimidin 4 yl) amino] cyclohexyl] methanesulfonamide’:ab,ti OR ‘n methyl 4 [n methyl n (7 hydropyrrolo [2, 3 d] pyrimidin 4 yl) amino] cyclohexanemethanesulfonamide’:ab,ti OR ‘n methyl 4 [n methyl n (7 pyrrolo [2, 3 d] pyrimidin 4 yl) amino] cyclohexanemethanesulfonamide’:ab,ti OR ‘n methyl 4 [n methyl n (7h pyrrolo [2, 3 d] pyrimidin 4 yl) amino] cyclohexanemethanesulfonamide’:ab,ti OR ‘n methyl [4 [methyl (7 hydropyrrolo [2, 3 d] pyrimidin 4 yl) amino] cyclohexyl] methanesulfonamide’:ab,ti OR ‘n methyl [4 [methyl (7 pyrrolo [2, 3 d] pyrimidin 4 yl) amino] cyclohexyl] methanesulfonamide’:ab,ti OR ‘n methyl [4 [methyl (7h pyrrolo [2, 3 d] pyrimidin 4 yl) amino] cyclohexyl] methanesulfonamide’:ab,ti OR ‘oclacitinib maleate’:ab,ti OR ‘pf 03394197’:ab,ti OR ‘pf 03394197 11’:ab,ti OR ‘pf 03394197-11’:ab,ti OR ‘pf03394197’:ab,ti OR ‘pf03394197 11’:ab,ti OR ‘pf03394197-11’:ab,ti OR ‘prolevare’:ab,ti OR ‘oclacitinib’:ab,ti OR ‘11 [2 (pyrrolidin 1 yl) ethoxy] 14, 19 dioxa 5, 7, 26 triazatetracyclo [19.3.1.1 (2, 6) .1 (8, 12)] heptacosa 1 (25), 2 (26), 3, 5, 8, 10, 12 (27), 16, 21, 23 decaene’:ab,ti OR ‘11 [2 (pyrrolidin 1 yl) ethoxy] 14, 19 dioxa 5, 7, 27 triazatetracyclo [19.3.1.1 (2, 6) .1 (8, 12)] heptacosa 1 (25), 2, 4, 6, 8, 10, 12 (26), 16, 21, 23 decaene’:ab,ti OR ‘enpaxiq’:ab,ti OR ‘epjevy’:ab,ti OR ‘onx 0803’:ab,ti OR ‘onx0803’:ab,ti OR ‘pacritinib citrate’:ab,ti OR ‘pacritinib hydrochloride’:ab,ti OR ‘sb 1518’:ab,ti OR ‘sb1518’:ab,ti OR ‘vonjo’:ab,ti OR ‘pacritinib’:ab,ti OR ‘4 [ (5 hydroxyadamantan 2 yl) amino] 1h pyrrolo [2, 3 b] pyridine 5 carboxamide’:ab,ti OR ‘4 [ (5 hydroxytricyclo [3.3.1.1 3, 7] dec 2 yl) amino] 1h pyrrolo [2, 3 b] pyridine 5 carboxamide’:ab,ti OR ‘asp 015k’:ab,ti OR ‘asp015k’:ab,ti OR ‘peficitinib hydrobromide’:ab,ti OR ‘peficitinib’:ab,ti OR ‘3 [4 (7h pyrrolo [2, 3 d] pyrimidin 4 yl) 1h pyrazol 1 yl] 3 cyclopentylpropanenitrile’:ab,ti OR ‘3 [4 (7h pyrrolo [2, 3 d] pyrimidin 4 yl) 1h pyrazol 1 yl] 3 cyclopentylpropanenitrile phosphate’:ab,ti OR ‘3 [4 (7h pyrrolo [2, 3 d] pyrimidin 4 yl) 1h pyrazolin 1 yl] 3 cyclopentylpropanenitrile’:ab,ti OR ‘3 [4 (7h pyrrolo [2, 3 d] pyrimidin 4 yl) 1h pyrazolin 1 yl] 3 cyclopentylpropanenitrile phosphate’:ab,ti OR ‘3 [4 (pyrrolo [2, 3 d] pyrimidin 4 yl) 1 pyrazolinyl] 3 cyclopentylpropanenitrile’:ab,ti OR ‘3 [4 (pyrrolo [2, 3 d] pyrimidin 4 yl) 1 pyrazolinyl] 3 cyclopentylpropanenitrile phosphate’:ab,ti OR ‘3 [4 (pyrrolo [2, 3 d] pyrimidin 4 yl) 1 pyrazolyl] 3 cyclopentylpropanenitrile’:ab,ti OR ‘3 [4 (pyrrolo [2, 3 d] pyrimidin 4 yl) 1 pyrazolyl] 3 cyclopentylpropanenitrile phosphate’:ab,ti OR ‘3 cyclopentyl 3 [4 (7h pyrrolo [2, 3 d] pyrimidin 4 yl) 1 pyrazolinyl] propanenitrile’:ab,ti OR ‘3 cyclopentyl 3 [4 (7h pyrrolo [2, 3 d] pyrimidin 4 yl) 1 pyrazolyl] propanenitrile’:ab,ti OR ‘3 cyclopentyl 3 [4 (7h pyrrolo [2, 3 d] pyrimidin 4 yl) 1h pyrazol 1 yl] propanenitrile’:ab,ti OR ‘3 cyclopentyl 3 [4 (7h pyrrolo [2, 3 d] pyrimidin 4 yl) pyrazol 1 yl] propanenitrile’:ab,ti OR ‘3 cyclopentyl 3 [4 (pyrrolo [2, 3 d] pyrimidin 4 yl) 1 pyrazolinyl] propanenitrile’:ab,ti OR ‘3 cyclopentyl 3 [4 (pyrrolo [2, 3 d] pyrimidin 4 yl) pyrazol 1 yl] propanenitrile’:ab,ti OR ‘beta cyclopentyl 4 (7h pyrrolo [2, 3 d] pyrimidin 4 yl) 1h pyrazole 1 propanenitrile’:ab,ti OR ‘beta cyclopentyl 4 (pyrrolo [2, 3 d] pyrimidin 4 yl) pyrazole 1 propanenitrile’:ab,ti OR ‘inc 424’:ab,ti OR ‘inc424’:ab,ti OR ‘incb 018424’:ab,ti OR ‘incb 18424’:ab,ti OR ‘incb 424’:ab,ti OR ‘incb018424’:ab,ti OR ‘incb18424’:ab,ti OR ‘incb424’:ab,ti OR ‘jakafi’:ab,ti OR ‘jakavi’:ab,ti OR ‘kks 278’:ab,ti OR ‘kks278’:ab,ti OR ‘opzelura’:ab,ti OR ‘ruxolitinib maleate’:ab,ti OR ‘ruxolitinib phosphate’:ab,ti OR ‘ruxolitinib’:ab,ti OR ‘1 cyanoacetyl 4 methyl n methyl n (1 hydropyrrolo [2, 3 d] pyrimidin 4 yl) 3 piperidinamine’:ab,ti OR ‘1 cyanoacetyl 4 methyl n methyl n (1 hydropyrrolo [2, 3 d] pyrimidin 4 yl) piperidine 3 amine’:ab,ti OR ‘1 cyanoacetyl 4 methyl n methyl n (1h pyrrolo [2, 3 d] pyrimidin 4 yl) 3 piperidinamine’:ab,ti OR ‘1 cyanoacetyl 4 methyl n methyl n (1h pyrrolo [2, 3 d] pyrimidin 4 yl) piperidine 3 amine’:ab,ti OR ‘3 [4 methyl 3 [methyl (7 hydropyrrolo [2, 3 d] pyrimidin 4 yl) amino] 1 piperidinyl] 3 oxopropanenitrile’:ab,ti OR ‘3 [4 methyl 3 [methyl (7 hydropyrrolo [2, 3 d] pyrimidin 4 yl) amino] 1 piperidyl] 3 oxopropanenitrile’:ab,ti OR ‘3 [4 methyl 3 [methyl (7 hydropyrrolo [2, 3 d] pyrimidin 4 yl) amino] piperidin 1 yl] 3 oxopropanenitrile’:ab,ti OR ‘3 [4 methyl 3 [methyl (7h pyrrolo [2, 3 d] pyrimidin 4 yl) amino] 1 piperidinyl] 3 oxopropanenitrile’:ab,ti OR ‘3 [4 methyl 3 [methyl (7h pyrrolo [2, 3 d] pyrimidin 4 yl) amino] 1 piperidyl] 3 oxopropanenitrile’:ab,ti OR ‘3 [4 methyl 3 [methyl (7h pyrrolo [2, 3 d] pyrimidin 4 yl) amino] piperidin 1 yl] 3 oxopropanenitrile’:ab,ti OR ‘4 [n [1 (2 cyano 1 oxoethyl) 4 methyl 3 piperidinyl] n methylamino] pyrrolo [2, 3 d] pyrimidine’:ab,ti OR ‘4 [n [1 (2 cyano 1 oxoethyl) 4 methyl 3 piperidyl] n methylamino] pyrrolo [2, 3 d] pyrimidine’:ab,ti OR ‘4 methyl 3 [methyl (7h pyrrolo [2, 3 d] pyrimidin 4 yl) amino] beta oxo 1 piperidinepropanenitrile’:ab,ti OR ‘cgb 500’:ab,ti OR ‘cgb500’:ab,ti OR ‘cp 690 550’:ab,ti OR ‘cp 690, 550’:ab,ti OR ‘cp 690550’:ab,ti OR ‘cp 690550 10’:ab,ti OR ‘cp 690550-10’:ab,ti OR ‘cp690 550’:ab,ti OR ‘cp690, 550’:ab,ti OR ‘cp690550’:ab,ti OR ‘cp690550 10’:ab,ti OR ‘cp690550-10’:ab,ti OR ‘jaquinus’:ab,ti OR ‘pgn 600’:ab,ti OR ‘pgn600’:ab,ti OR ‘prd 4862257’:ab,ti OR ‘prd4862257’:ab,ti OR ‘ro 5169503’:ab,ti OR ‘ro5169503’:ab,ti OR ‘tasocitinib’:ab,ti OR ‘tasocitinib citrate’:ab,ti OR ‘tofacitinib citrate’:ab,ti OR ‘xeljanz’:ab,ti OR ‘xeljanz xr’:ab,ti OR ‘tofacitinib’:ab,ti OR ‘3 ethyl 4 (1, 5, 7, 10 tetrazatricyclo [7.3.0.0 (2, 6)] dodeca 2 (6), 3, 7, 9, 11 pentaen 12 yl) n (2, 2, 2 trifluoroethyl) pyrrolidine 1 carboxamide’:ab,ti OR ‘3 ethyl 4 (3h imidazo [1, 2 a] pyrrolo [2, 3 e] pyrazin 8 yl) n (2, 2, 2 trifluoroethyl) 1 pyrrolidinecarboxamide’:ab,ti OR ‘3 ethyl 4 (3h imidazo [1, 2 a] pyrrolo [2, 3 e] pyrazin 8 yl) n (2, 2, 2 trifluoroethyl) 1 pyrrolidinecarboxamide 2, 3 dihydroxybutanedioate’:ab,ti OR ‘3 ethyl 4 (3h imidazo [1, 2 a] pyrrolo [2, 3 e] pyrazin 8 yl) n (2, 2, 2 trifluoroethyl) 1 pyrrolidinecarboxamide tartrate’:ab,ti OR ‘3 ethyl 4 (3h imidazo [1, 2 a] pyrrolo [2, 3 e] pyrazin 8 yl) n (2, 2, 2 trifluoroethyl) pyrrolidine 1 carboxamide’:ab,ti OR ‘3 ethyl 4 (3h imidazo [1, 2 a] pyrrolo [2, 3 e] pyrazin 8 yl) n (2, 2, 2 trifluoroethyl) pyrrolidine 1 carboxamide 2, 3 dihydroxybutanedioate’:ab,ti OR ‘3 ethyl 4 (3h imidazo [1, 2 a] pyrrolo [2, 3 e] pyrazin 8 yl) n (2, 2, 2 trifluoroethyl) pyrrolidine 1 carboxamide tartrate’:ab,ti OR ‘abt 494’:ab,ti OR ‘abt494’:ab,ti OR ‘rinvoq’:ab,ti OR ‘upadacitinib 2, 3 dihydroxybutanedioate’:ab,ti OR ‘upadacitinib hemihydrate’:ab,ti OR ‘upadacitinib hydrate’:ab,ti OR ‘upadacitinib tartrate’:ab,ti OR ‘upadacitinib’:ab,ti

3. #1 AND #2

**Scopus**

1. TITLE-ABS-KEY(“Sweet syndrome” OR “Syndrome, Sweet” OR “Dermatosis, Neutrophilic, Febrile, Acute” OR “Acute Febrile Neutrophilic Dermatosis” OR “Sweet's Syndrome” OR “Sweets Syndrome” OR “Syndrome, Sweet's” OR “Gomm Button Disease” OR “Disease, Gomm Button” OR “Gomm-Button Disease” OR “Disease, Gomm-Button” OR “Neutrophilic Dermatosis, Acute Febrile” OR “acute febrile neutrophil dermatosis” OR “acute febrile neutrophilic dermatitis” OR “dermatosis, acute febrile neutrophilic” OR “Gomm-Button disease” OR “neutrophilic dermatosis, acute febrile” OR “dermatitis ulcerosa” OR “ulcerative dermatitis” OR “pyodermatitis” OR “pyodermia” OR “pyodermitis” OR “pyroderma” OR “pyoderma”)

2. (TITLE-ABS-KEY("Janus Kinase Inhibitors")) OR (TITLE-ABS-KEY("Inhibitors, Janus Kinase")) OR (TITLE-ABS-KEY("Kinase Inhibitors, Janus")) OR (TITLE-ABS-KEY("JAK Inhibitors")) OR (TITLE-ABS-KEY("Inhibitors, JAK")) OR (TITLE-ABS-KEY("Janus Kinase Inhibitor")) OR (TITLE-ABS-KEY("Inhibitor, Janus Kinase")) OR (TITLE-ABS-KEY("Kinase Inhibitor, Janus")) OR (TITLE-ABS-KEY("JAK Inhibitor")) OR (TITLE-ABS-KEY("Inhibitor, JAK")) OR (TITLE-ABS-KEY("JAK inhibitor")) OR (TITLE-ABS-KEY("Janus kinase inhibitors")) OR (TITLE-ABS-KEY("Janus tyrosine kinase inhibitor")) OR (TITLE-ABS-KEY("Janus kinase inhibitor")) OR (TITLE-ABS-KEY("cibinqo")) OR (TITLE-ABS-KEY("pf 04965842")) OR (TITLE-ABS-KEY("pf04965842")) OR (TITLE-ABS-KEY("abrocitinib")) OR (TITLE-ABS-KEY("incb 028050")) OR (TITLE-ABS-KEY("incb 28050")) OR (TITLE-ABS-KEY("incb028050")) OR (TITLE-ABS-KEY("ly 3009104")) OR (TITLE-ABS-KEY("ly3009104")) OR (TITLE-ABS-KEY("olumiant")) OR (TITLE-ABS-KEY("baricitinib")) OR (TITLE-ABS-KEY("jte 052")) OR (TITLE-ABS-KEY("jte 052a")) OR (TITLE-ABS-KEY("jte052")) OR (TITLE-ABS-KEY("leo 124249")) OR (TITLE-ABS-KEY("leo124249")) OR (TITLE-ABS-KEY("delgocitinib")) OR (TITLE-ABS-KEY("fedratinib dihydrochloride")) OR (TITLE-ABS-KEY("fedratinib dihydrochloride monohydrate")) OR (TITLE-ABS-KEY("fedratinib hydrochloride")) OR (TITLE-ABS-KEY("inrebic")) OR (TITLE-ABS-KEY("sar302503")) OR (TITLE-ABS-KEY("tg 101348")) OR (TITLE-ABS-KEY("tg101348")) OR (TITLE-ABS-KEY("fedratinib")) OR (TITLE-ABS-KEY("filgotinib 2 butenedioate")) OR (TITLE-ABS-KEY("filgotinib hydrochloride")) OR (TITLE-ABS-KEY("filgotinib maleate")) OR (TITLE-ABS-KEY("glpg 0634")) OR (TITLE-ABS-KEY("glpg0634")) OR (TITLE-ABS-KEY("gs 6034")) OR (TITLE-ABS-KEY("gs6034")) OR (TITLE-ABS-KEY("jyseleca")) OR (TITLE-ABS-KEY("apoquel")) OR (TITLE-ABS-KEY("oclacitinib maleate")) OR (TITLE-ABS-KEY("oclacitinib")) OR (TITLE-ABS-KEY("onx 0803")) OR (TITLE-ABS-KEY("pacritinib citrate")) OR (TITLE-ABS-KEY("pacritinib hydrochloride")) OR (TITLE-ABS-KEY("sb 1518")) OR (TITLE-ABS-KEY("sb1518")) OR (TITLE-ABS-KEY("vonjo")) OR (TITLE-ABS-KEY("pacritinib")) OR (TITLE-ABS-KEY("asp015k")) OR (TITLE-ABS-KEY("peficitinib hydrobromide")) OR (TITLE-ABS-KEY("peficitinib")) OR (TITLE-ABS-KEY("incb 18424")) OR (TITLE-ABS-KEY("incb 424")) OR (TITLE-ABS-KEY("incb018424")) OR (TITLE-ABS-KEY("incb18424")) OR (TITLE-ABS-KEY("jakafi")) OR (TITLE-ABS-KEY("jakavi")) OR (TITLE-ABS-KEY("kks 278")) OR (TITLE-ABS-KEY("opzelura")) OR (TITLE-ABS-KEY("ruxolitinib maleate")) OR (TITLE-ABS-KEY("ruxolitinib phosphate")) OR (TITLE-ABS-KEY("ruxolitinib")) OR (TITLE-ABS-KEY("cgb 500")) OR (TITLE-ABS-KEY("cgb500")) OR (TITLE-ABS-KEY("cp 690 550")) OR (TITLE-ABS-KEY("cp 690, 550")) OR (TITLE-ABS-KEY("cp 690550")) OR (TITLE-ABS-KEY("cp 690550 10")) OR (TITLE-ABS-KEY("cp690 550")) OR (TITLE-ABS-KEY("cp690, 550")) OR (TITLE-ABS-KEY("cp690550")) OR (TITLE-ABS-KEY("cp690550 10")) OR (TITLE-ABS-KEY("pgn 600")) OR (TITLE-ABS-KEY("tasocitinib")) OR (TITLE-ABS-KEY("tasocitinib citrate")) OR (TITLE-ABS-KEY("tofacitinib citrate")) OR (TITLE-ABS-KEY("xeljanz")) OR (TITLE-ABS-KEY("xeljanz xr")) OR (TITLE-ABS-KEY("tofacitinib")) OR (TITLE-ABS-KEY("abt 494")) OR (TITLE-ABS-KEY("abt494")) OR (TITLE-ABS-KEY("rinvoq")) OR (TITLE-ABS-KEY("upadacitinib 2, 3 dihydroxybutanedioate")) OR (TITLE-ABS-KEY("upadacitinib hemihydrate")) OR (TITLE-ABS-KEY("upadacitinib hydrate")) OR (TITLE-ABS-KEY("upadacitinib tartrate")) OR (TITLE-ABS-KEY("upadacitinib"))

3. #1 AND #2

**Web of Science**

1. TS=(“Sweet syndrome” OR “Syndrome, Sweet” OR “Dermatosis, Neutrophilic, Febrile, Acute” OR “Acute Febrile Neutrophilic Dermatosis” OR “Sweet's Syndrome” OR “Sweets Syndrome” OR “Syndrome, Sweet's” OR “Gomm Button Disease” OR “Disease, Gomm Button” OR “Gomm-Button Disease” OR “Disease, Gomm-Button” OR “Neutrophilic Dermatosis, Acute Febrile” OR “acute febrile neutrophil dermatosis” OR “acute febrile neutrophilic dermatitis” OR “dermatosis, acute febrile neutrophilic” OR “Gomm-Button disease” OR “neutrophilic dermatosis, acute febrile” OR “dermatitis ulcerosa” OR “ulcerative dermatitis” OR “pyodermatitis” OR “pyodermia” OR “pyodermitis” OR “pyroderma” OR “pyoderma”)

2. TS=(“Janus Kinase Inhibitors” OR “Inhibitors, Janus Kinase” OR “Kinase Inhibitors, Janus” OR “JAK Inhibitors” OR “Inhibitors, JAK” OR “Janus Kinase Inhibitor” OR “Inhibitor, Janus Kinase” OR “Kinase Inhibitor, Janus” OR “JAK Inhibitor” OR “Inhibitor, JAK” OR “JAK inhibitor” OR “Janus kinase inhibitors” OR “Janus tyrosine kinase inhibitor” OR “Janus kinase inhibitor” OR “cibinqo” OR “n [3 (methyl 7 hydropyrrolo [2, 3 d] pyrimidin 4 ylamino) cyclobutyl] 1 propanesulfonamide” OR “n [3 (methyl 7 hydropyrrolo [2, 3 d] pyrimidin 4 ylamino) cyclobutyl] propane 1 sulfonamide” OR “n [3 (methyl 7h pyrrolo [2, 3 d] pyrimidin 4 ylamino) cyclobutyl] 1 propanesulfonamide” OR “n [3 (methyl 7h pyrrolo [2, 3 d] pyrimidin 4 ylamino) cyclobutyl] propane 1 sulfonamide” OR “n [3 (methylpyrrolo [2, 3 d] pyrimidin 4 ylamino) cyclobutyl] 1 propanesulfonamide” OR “n [3 (methylpyrrolo [2, 3 d] pyrimidin 4 ylamino) cyclobutyl] propane 1 sulfonamide” OR “n [3 [methyl (7 hydropyrrolo [2, 3 d] pyrimidin 4 yl) amino] cyclobutyl] 1 propanesulfonamide” OR “n [3 [methyl (7 hydropyrrolo [2, 3 d] pyrimidin 4 yl) amino] cyclobutyl] propane 1 sulfonamide” OR “n [3 [methyl (7h pyrrolo [2, 3 d] pyrimidin 4 yl) amino] cyclobutyl] 1 propanesulfonamide” OR “n [3 [methyl (7h pyrrolo [2, 3 d] pyrimidin 4 yl) amino] cyclobutyl] propane 1 sulfonamide” OR “pf 04965842” OR “pf 4965842” OR “pf04965842” OR “pf4965842” OR “abrocitinib” OR “1 (ethylsulfonyl) 3 [4 (7h pyrrolo [2, 3 d] pyrimidin 4 yl) 1h 1 pyrazolyl] 3 azetidineacetonitrile” OR “1 (ethylsulfonyl) 3 [4 (7h pyrrolo [2, 3 d] pyrimidin 4 yl) 1h pyrazol 1 yl] 3 azetidineacetonitrile” OR “2 (3 (4 (3h pyrrolo [2, 3 d] pyrimidin 4 yl) 1h 1 pyrazolyl) 1 (ethylsulfonyl) 3 azetidinyl) acetonitrile” OR “2 (3 (4 (3h pyrrolo [2, 3 d] pyrimidin 4 yl) 1h 1 pyrazolyl) 1 (ethylsulfonyl) 3 azetidyl) acetonitrile” OR “2 (3 (4 (3h pyrrolo [2, 3 d] pyrimidin 4 yl) 1h pyrazol 1 yl) 1 (ethylsulfonyl) azetidin 3 yl) acetonitrile” OR “[1 (ethanesulfonyl) 3 [4 (7 hydropyrrolo [2, 3 d] pyrimidin 4 yl) 1 hydropyrazol 1 yl] azetidin 3 yl] ethanenitrile” OR “[1 (ethanesulfonyl) 3 [4 (7h pyrrolo [2, 3 d] pyrimidin 4 yl) 1h 1 pyrazolyl] 3 azetidinyl] ethanenitrile” OR “[1 (ethanesulfonyl) 3 [4 (7h pyrrolo [2, 3 d] pyrimidin 4 yl) 1h 1 pyrazolyl] 3 azetidyl] ethanenitrile” OR “[1 (ethanesulfonyl) 3 [4 (7h pyrrolo [2, 3 d] pyrimidin 4 yl) 1h pyrazol 1 yl] azetidin 3 yl] ethanenitrile” OR “[1 (ethylsulfonyl) 3 [4 (1h pyrrolo [2, 3 d] pyrimidin 4 yl) 1h 1 pyrazolyl] 3 azetidinyl] acetonitrile” OR “[1 (ethylsulfonyl) 3 [4 (1h pyrrolo [2, 3 d] pyrimidin 4 yl) 1h 1 pyrazolyl] 3 azetidyl] acetonitrile” OR “[1 (ethylsulfonyl) 3 [4 (1h pyrrolo [2, 3 d] pyrimidin 4 yl) 1h pyrazol 1 yl] 3 azetidinyl] acetonitrile” OR “[1 (ethylsulfonyl) 3 [4 (7h pyrrolo [2, 3 d] pyrimidin 4 yl) 1h 1 pyrazolyl] 3 azetidinyl] ethanenitrile” OR “[1 (ethylsulfonyl) 3 [4 (7h pyrrolo [2, 3 d] pyrimidin 4 yl) 1h 1 pyrazolyl] 3 azetidyl] ethanenitrile” OR “[1 (ethylsulfonyl) 3 [4 (7h pyrrolo [2, 3 d] pyrimidin 4 yl) 1h pyrazol 1 yl] azetidin 3 yl] ethanenitrile” OR “incb 028050” OR “incb 28050” OR “incb028050” OR “incb28050” OR “ly 3009104” OR “ly3009104” OR “olumiant” OR “baricitinib” OR “3 [3 methyl 6 (7h pyrrolo [2, 3 d] pyrimidin 4 yl) 1, 6 diazaspiro [3.4] octan 1 yl] 3 oxopropanenitrile” OR “jte 052” OR “jte 052a” OR “jte052” OR “jte052a” OR “leo 124249” OR “leo 124249a” OR “leo124249” OR “leo124249a” OR “delgocitinib” OR “fedratinib dihydrochloride” OR “fedratinib dihydrochloride monohydrate” OR “fedratinib hydrochloride” OR “inrebic” OR “n (1, 1 dimethylethyl) 3 [5 methyl 2 [4 (2 pyrrolidin 1 ylethoxy) phenylamino] pyrimidin 4 ylamino] benzenesulfonamide” OR “n (1, 1 dimethylethyl) 3 [5 methyl 2 [4 [2 (1 pyrrolidinyl) ethoxy] phenylamino] 4 pyrimidinylamino] benzenesulfonamide” OR “n (1, 1 dimethylethyl) 3 [5 methyl 2 [4 [2 (1 pyrrolidyl) ethoxy] phenylamino] 4 pyrimidylamino] benzenesulfonamide” OR “n (1, 1 dimethylethyl) 3 [ [5 methyl 2 [4 [2 (pyrrolidin 1 yl) ethoxy] anilino] pyrimidin 4 yl] amino] benzenesulfonamide” OR “n (1, 1 dimethylethyl) 3 [ [5 methyl 2 [ [4 (2 pyrrolidin 1 ylethoxy) phenyl] amino] pyrimidin 4 yl] amino] benzenesulfonamide” OR “n (1, 1 dimethylethyl) 3 [ [5 methyl 2 [ [4 [2 (1 pyrrolidinyl) ethoxy] phenyl] amino] 4 pyrimidinyl] amino] benzenesulfonamide” OR “n (1, 1 dimethylethyl) 3 [ [5 methyl 2 [ [4 [2 (1 pyrrolidyl) ethoxy] phenyl] amino] 4 pyrimidyl] amino] benzenesulfonamide” OR “n (1, 1 dimethylethyl) 3 [ [5 methyl 2 [ [4 [2 (pyrrolidin 1 yl) ethoxy] phenyl] amino] pyrimidin 4 yl] amino] benzenesulfonamide” OR “n tert butyl 3 [5 methyl 2 [4 (2 pyrrolidin 1 ylethoxy) phenylamino] pyrimidin 4 ylamino] benzenesulfonamide” OR “n tert butyl 3 [5 methyl 2 [4 [2 (1 pyrrolidinyl) ethoxy] phenylamino] 4 pyrimidinylamino] benzenesulfonamide” OR “n tert butyl 3 [5 methyl 2 [4 [2 (1 pyrrolidyl) ethoxy] phenylamino] 4 pyrimidylamino] benzenesulfonamide” OR “n tert butyl 3 [ [5 methyl 2 [4 [2 (1 pyrrolidinyl) ethoxy] anilino] 4 pyrimidinyl] amino] benzenesulfonamide” OR “n tert butyl 3 [ [5 methyl 2 [4 [2 (1 pyrrolidyl) ethoxy] anilino] 4 pyrimidyl] amino] benzenesulfonamide” OR “n tert butyl 3 [ [5 methyl 2 [4 [2 (pyrrolidin 1 yl) ethoxy] anilino] pyrimidin 4 yl] amino] benzenesulfonamide” OR “n tert butyl 3 [ [5 methyl 2 [ [4 (2 pyrrolidin 1 ylethoxy) phenyl] amino] pyrimidin 4 yl] amino] benzenesulfonamide” OR “sar 302503” OR “sar 302503a” OR “sar302503” OR “sar302503a” OR “tg 101348” OR “tg101348” OR “fedratinib” OR “filgotinib 2 butenedioate” OR “filgotinib hydrochloride” OR “filgotinib maleate” OR “g 146034” OR “g 146034 101” OR “g 146034-101” OR “g146034” OR “g146034 101” OR “g146034-101” OR “glpg 0634” OR “glpg0634” OR “gs 6034” OR “gs6034” OR “jyseleca” OR “n [5 [4 (1, 1 dioxothiomorpholinomethyl) phenyl] 1, 2, 4 triazolo [1, 5 a] pyridin 2 yl] cyclopropanecarboxamide” OR “n [5 [4 (1, 1 dioxothiomorpholinomethyl) phenyl] 1, 2, 4 triazolo [1, 5 a] pyridin 2 yl] cyclopropanecarboxamide 2 butenedioate” OR “n [5 [4 (1, 1 dioxothiomorpholinomethyl) phenyl] 1, 2, 4 triazolo [1, 5 a] pyridin 2 yl] cyclopropanecarboxamide but 2 enedioate” OR “n [5 [4 [ (1, 1 dioxido 4 thiomorpholinyl) methyl] phenyl] 1, 2, 4 triazolo [1, 5 a] pyridin 2 yl] cyclopropanecarboxamide” OR “n [5 [4 [ (1, 1 dioxido 4 thiomorpholinyl) methyl] phenyl] 1, 2, 4 triazolo [1, 5 a] pyridin 2 yl] cyclopropanecarboxamide 2 butenedioate” OR “n [5 [4 [ (1, 1 dioxidothiomorpholin 4 yl) methyl] phenyl] [1, 2, 4] triazolo [1, 5 a] pyridin 2 yl] cyclopropanecarboxamide” OR “n [5 [4 [ (1, 1 dioxo 1, 4 thiazinan 4 yl) methyl] phenyl] [1, 2, 4] triazolo [1, 5 a] pyridin 2 yl] cyclopropanecarboxamide” OR “n [5 [4 [ (1, 1 dioxothiomorpholin 4 yl) methyl] phenyl] 1, 2, 4 triazolo [1, 5 a] pyridin 2 yl] cyclopropanecarboxamide” OR “n [5 [4 [ (1, 1 dioxothiomorpholin 4 yl) methyl] phenyl] 1, 2, 4 triazolo [1, 5 a] pyridin 2 yl] cyclopropanecarboxamide but 2 enedioate” OR “n [5 [4 [ (1, 1 dioxothiomorpholin 4 yl) methyl] phenyl] [1, 2, 4] triazolo [1, 5 a] pyridin 2 yl] cyclopropanecarboxamide” OR “n [5 [4 [ (1, 1 dioxothiomorpholin 4 yl) methyl] phenyl] [1, 2, 4] triazolo [1, 5 a] pyridin 2 yl] cyclopropanecarboxamide but 2 enedioate” OR “filgotinib” OR “apoquel” OR “n methyl 1 [4 [methyl (7 hydropyrrolo [2, 3 d] pyrimidin 4 yl) amino] cyclohexyl] methanesulfonamide” OR “n methyl 1 [4 [methyl (7h pyrrolo [2, 3 d] pyrimidin 4 yl) amino] cyclohexyl] methanesulfonamide” OR “n methyl 4 [n methyl n (7 hydropyrrolo [2, 3 d] pyrimidin 4 yl) amino] cyclohexanemethanesulfonamide” OR “n methyl 4 [n methyl n (7 pyrrolo [2, 3 d] pyrimidin 4 yl) amino] cyclohexanemethanesulfonamide” OR “n methyl 4 [n methyl n (7h pyrrolo [2, 3 d] pyrimidin 4 yl) amino] cyclohexanemethanesulfonamide” OR “n methyl [4 [methyl (7 hydropyrrolo [2, 3 d] pyrimidin 4 yl) amino] cyclohexyl] methanesulfonamide” OR “n methyl [4 [methyl (7 pyrrolo [2, 3 d] pyrimidin 4 yl) amino] cyclohexyl] methanesulfonamide” OR “n methyl [4 [methyl (7h pyrrolo [2, 3 d] pyrimidin 4 yl) amino] cyclohexyl] methanesulfonamide” OR “oclacitinib maleate” OR “pf 03394197” OR “pf 03394197 11” OR “pf 03394197-11” OR “pf03394197” OR “pf03394197 11” OR “pf03394197-11” OR “prolevare” OR “oclacitinib” OR “11 [2 (pyrrolidin 1 yl) ethoxy] 14, 19 dioxa 5, 7, 26 triazatetracyclo [19.3.1.1 (2, 6) .1 (8, 12)] heptacosa 1 (25), 2 (26), 3, 5, 8, 10, 12 (27), 16, 21, 23 decaene” OR “11 [2 (pyrrolidin 1 yl) ethoxy] 14, 19 dioxa 5, 7, 27 triazatetracyclo [19.3.1.1 (2, 6) .1 (8, 12)] heptacosa 1 (25), 2, 4, 6, 8, 10, 12 (26), 16, 21, 23 decaene” OR “enpaxiq” OR “epjevy” OR “onx 0803” OR “onx0803” OR “pacritinib citrate” OR “pacritinib hydrochloride” OR “sb 1518” OR “sb1518” OR “vonjo” OR “pacritinib” OR “4 [ (5 hydroxyadamantan 2 yl) amino] 1h pyrrolo [2, 3 b] pyridine 5 carboxamide” OR “4 [ (5 hydroxytricyclo [3.3.1.1 3, 7] dec 2 yl) amino] 1h pyrrolo [2, 3 b] pyridine 5 carboxamide” OR “asp 015k” OR “asp015k” OR “peficitinib hydrobromide” OR “peficitinib” OR “3 [4 (7h pyrrolo [2, 3 d] pyrimidin 4 yl) 1h pyrazol 1 yl] 3 cyclopentylpropanenitrile” OR “3 [4 (7h pyrrolo [2, 3 d] pyrimidin 4 yl) 1h pyrazol 1 yl] 3 cyclopentylpropanenitrile phosphate” OR “3 [4 (7h pyrrolo [2, 3 d] pyrimidin 4 yl) 1h pyrazolin 1 yl] 3 cyclopentylpropanenitrile” OR “3 [4 (7h pyrrolo [2, 3 d] pyrimidin 4 yl) 1h pyrazolin 1 yl] 3 cyclopentylpropanenitrile phosphate” OR “3 [4 (pyrrolo [2, 3 d] pyrimidin 4 yl) 1 pyrazolinyl] 3 cyclopentylpropanenitrile” OR “3 [4 (pyrrolo [2, 3 d] pyrimidin 4 yl) 1 pyrazolinyl] 3 cyclopentylpropanenitrile phosphate” OR “3 [4 (pyrrolo [2, 3 d] pyrimidin 4 yl) 1 pyrazolyl] 3 cyclopentylpropanenitrile” OR “3 [4 (pyrrolo [2, 3 d] pyrimidin 4 yl) 1 pyrazolyl] 3 cyclopentylpropanenitrile phosphate” OR “3 cyclopentyl 3 [4 (7h pyrrolo [2, 3 d] pyrimidin 4 yl) 1 pyrazolinyl] propanenitrile” OR “3 cyclopentyl 3 [4 (7h pyrrolo [2, 3 d] pyrimidin 4 yl) 1 pyrazolyl] propanenitrile” OR “3 cyclopentyl 3 [4 (7h pyrrolo [2, 3 d] pyrimidin 4 yl) 1h pyrazol 1 yl] propanenitrile” OR “3 cyclopentyl 3 [4 (7h pyrrolo [2, 3 d] pyrimidin 4 yl) pyrazol 1 yl] propanenitrile” OR “3 cyclopentyl 3 [4 (pyrrolo [2, 3 d] pyrimidin 4 yl) 1 pyrazolinyl] propanenitrile” OR “3 cyclopentyl 3 [4 (pyrrolo [2, 3 d] pyrimidin 4 yl) pyrazol 1 yl] propanenitrile” OR “beta cyclopentyl 4 (7h pyrrolo [2, 3 d] pyrimidin 4 yl) 1h pyrazole 1 propanenitrile” OR “beta cyclopentyl 4 (pyrrolo [2, 3 d] pyrimidin 4 yl) pyrazole 1 propanenitrile” OR “inc 424” OR “inc424” OR “incb 018424” OR “incb 18424” OR “incb 424” OR “incb018424” OR “incb18424” OR “incb424” OR “jakafi” OR “jakavi” OR “kks 278” OR “kks278” OR “opzelura” OR “ruxolitinib maleate” OR “ruxolitinib phosphate” OR “ruxolitinib” OR “1 cyanoacetyl 4 methyl n methyl n (1 hydropyrrolo [2, 3 d] pyrimidin 4 yl) 3 piperidinamine” OR “1 cyanoacetyl 4 methyl n methyl n (1 hydropyrrolo [2, 3 d] pyrimidin 4 yl) piperidine 3 amine” OR “1 cyanoacetyl 4 methyl n methyl n (1h pyrrolo [2, 3 d] pyrimidin 4 yl) 3 piperidinamine” OR “1 cyanoacetyl 4 methyl n methyl n (1h pyrrolo [2, 3 d] pyrimidin 4 yl) piperidine 3 amine” OR “3 [4 methyl 3 [methyl (7 hydropyrrolo [2, 3 d] pyrimidin 4 yl) amino] 1 piperidinyl] 3 oxopropanenitrile” OR “3 [4 methyl 3 [methyl (7 hydropyrrolo [2, 3 d] pyrimidin 4 yl) amino] 1 piperidyl] 3 oxopropanenitrile” OR “3 [4 methyl 3 [methyl (7 hydropyrrolo [2, 3 d] pyrimidin 4 yl) amino] piperidin 1 yl] 3 oxopropanenitrile” OR “3 [4 methyl 3 [methyl (7h pyrrolo [2, 3 d] pyrimidin 4 yl) amino] 1 piperidinyl] 3 oxopropanenitrile” OR “3 [4 methyl 3 [methyl (7h pyrrolo [2, 3 d] pyrimidin 4 yl) amino] 1 piperidyl] 3 oxopropanenitrile” OR “3 [4 methyl 3 [methyl (7h pyrrolo [2, 3 d] pyrimidin 4 yl) amino] piperidin 1 yl] 3 oxopropanenitrile” OR “4 [n [1 (2 cyano 1 oxoethyl) 4 methyl 3 piperidinyl] n methylamino] pyrrolo [2, 3 d] pyrimidine” OR “4 [n [1 (2 cyano 1 oxoethyl) 4 methyl 3 piperidyl] n methylamino] pyrrolo [2, 3 d] pyrimidine” OR “4 methyl 3 [methyl (7h pyrrolo [2, 3 d] pyrimidin 4 yl) amino] beta oxo 1 piperidinepropanenitrile” OR “cgb 500” OR “cgb500” OR “cp 690 550” OR “cp 690, 550” OR “cp 690550” OR “cp 690550 10” OR “cp 690550-10” OR “cp690 550” OR “cp690, 550” OR “cp690550” OR “cp690550 10” OR “cp690550-10” OR “jaquinus” OR “pgn 600” OR “pgn600” OR “prd 4862257” OR “prd4862257” OR “ro 5169503” OR “ro5169503” OR “tasocitinib” OR “tasocitinib citrate” OR “tofacitinib citrate” OR “xeljanz” OR “xeljanz xr” OR “tofacitinib” OR “3 ethyl 4 (1, 5, 7, 10 tetrazatricyclo [7.3.0.0 (2, 6)] dodeca 2 (6), 3, 7, 9, 11 pentaen 12 yl) n (2, 2, 2 trifluoroethyl) pyrrolidine 1 carboxamide” OR “3 ethyl 4 (3h imidazo [1, 2 a] pyrrolo [2, 3 e] pyrazin 8 yl) n (2, 2, 2 trifluoroethyl) 1 pyrrolidinecarboxamide” OR “3 ethyl 4 (3h imidazo [1, 2 a] pyrrolo [2, 3 e] pyrazin 8 yl) n (2, 2, 2 trifluoroethyl) 1 pyrrolidinecarboxamide 2, 3 dihydroxybutanedioate” OR “3 ethyl 4 (3h imidazo [1, 2 a] pyrrolo [2, 3 e] pyrazin 8 yl) n (2, 2, 2 trifluoroethyl) 1 pyrrolidinecarboxamide tartrate” OR “3 ethyl 4 (3h imidazo [1, 2 a] pyrrolo [2, 3 e] pyrazin 8 yl) n (2, 2, 2 trifluoroethyl) pyrrolidine 1 carboxamide” OR “3 ethyl 4 (3h imidazo [1, 2 a] pyrrolo [2, 3 e] pyrazin 8 yl) n (2, 2, 2 trifluoroethyl) pyrrolidine 1 carboxamide 2, 3 dihydroxybutanedioate” OR “3 ethyl 4 (3h imidazo [1, 2 a] pyrrolo [2, 3 e] pyrazin 8 yl) n (2, 2, 2 trifluoroethyl) pyrrolidine 1 carboxamide tartrate” OR “abt 494” OR “abt494” OR “rinvoq” OR “upadacitinib 2, 3 dihydroxybutanedioate” OR “upadacitinib hemihydrate” OR “upadacitinib hydrate” OR “upadacitinib tartrate” OR “upadacitinib”)

3. #1 AND #2

Date of search July 25, 2025

Number of records

PubMed 65

Embase 299

Scopus 264

Web of Science 61

Total 689

Duplicates 347

Non duplicate 342
