# Supplementary material for: JAK Inhibitors for Treatment of Pyoderma Gangrenosum and Sweet Syndrome: A Systematic Review of Published Case Reports
Source: Dermatol Res Pract. 2026 Jul 18;2026:7086209. doi: 10.1155/drp/7086209 (PMC13379896; doi:10.1155/drp/7086209)
Supplement: Supplementary file 2 — Supporting Information 2 Supporting file 2 shows quality assessment of included studies. [file DRP-2026-7086209-s003.docx]

Risk of Bias assessment for case reports and case series (Good, Fair, Poor)

| # | Study | Selection | Ascertainment | | Causality | | | | Reporting | Overall |
| --- | --- | --- | --- | --- | --- | --- | --- | --- | --- | --- |
|  |  | Q1 | Q2 | Q3 | Q4 | Q5 | Q6 | Q7 | Q8 |  |
| 1 | Estrella 2025 | Yes | Yes | Yes | Yes | NA | No | Yes | Yes | Good |
| 2 | Yeo 2024 | Yes | Yes | No | Yes | NA | No | No | Yes | Fair |
| 3 | Chen 2024 | Yes | Yes | Yes | No | NA | No | Yes | Yes | Good |
| 4 | Wang 2024 | Yes | Yes | Yes | Yes | NA | No | Yes | Yes | Good |
| 5 | Ito 2024 | Yes | No | No | Yes | NA | No | Yes | No | Poor |
| 6 | Grise 2024 | Yes | Yes | Yes | Yes | Yes | Yes | Yes | Yes | Good |
| 7 | Choe 2024 | Yes | No | No | Yes | NA | No | Yes | No | Poor |
| 8 | Bechard 2024 | Yes | Yes | Yes | No | NA | No | Yes | Yes | Good |
| 9 | Kim 2023 | Yes | Yes | No | No | NA | No | No | Yes | Fair |
| 10 | Castro 2023 | Yes | Yes | Yes | Yes | NA | Yes | Yes | Yes | Good |
| 11 | Sitaru 2022 | Yes | Yes | Yes | No | NA | No | Yes | Yes | Good |
| 12 | Scheinberg 2021 | Yes | Yes | Yes | Yes | NA | No | Yes | Yes | Good |
| 13 | Shibuta 2025 | Yes | Yes | No | No | NA | Yes | Yes | Yes | Fair |
| 14 | Nasifoglu 2018 | Yes | No | Yes | Yes | NA | No | Yes | No | Poor |
| 15 | Shanmugam 2013 | Yes | Yes | Yes | No | NA | No | Yes | Yes | Good |
| 16 | Xiao 2024 | Yes | Yes | No | Yes | NA | No | Yes | Yes | Fair |
| 17 | Narula 2024 | Yes | Yes | Yes | Yes | NA | Yes | No | Yes | Good |
| 18 | Köken Avşar2024 | Yes | Yes | Yes | No | NA | No | Yes | Yes | Fair |
| 19 | Bhowmick2023 | Yes | Yes | Yes | No | NA | No | Yes | Yes | Good |
| 20 | Sathyanarayana2023 | NA | Yes | Yes | Yes | NA | No | Yes | Yes | Good |
| 21 | Bhadresha2021 | Yes | Yes | Yes | No | NA | No | Yes | Yes | Fair |
| 22 | Olavarría2021 | Yes | Yes | Yes | No | NA | No | No | Yes | Fair |
| 23 | Sedano 2021 | Yes | Yes | No | Yes | NA | No | No | Yes | Fair |
| 24 | Choi 2020 | Yes | Yes | Yes | Yes | NA | Yes | Yes | Yes | Good |
| 25 | Gregory2019 | Yes | Yes | Yes | No | NA | No | Yes | Yes | Good |
| 26 | Kochar2019 | NA | Yes | Yes | No | NA | No | Yes | Yes | Good |
| 27 | Korytnikova2025 | Yes | Yes | Yes | No | NA | No | Yes | Yes | Good |
| 28 | Taha2025 | Yes | Yes | Yes | Yes | NA | Yes | Yes | Yes | Good |
| 29 | Patel2025 | Yes | Yes | Yes | Yes | NA | No | Yes | No | Poor |
| 30 | He2025 | Yes | Yes | Yes | No | NA | No | Yes | Yes | Good |
| 31 | Prieto Jimenez2024 | Yes | Yes | Yes | Yes | NA | No | Yes | Yes | Good |
| 32 | Zaher2024 | Yes | Yes | Yes | Yes | NA | No | Yes | Yes | Good |
| 33 | Park2024 | Yes | Yes | Yes | No | NA | No | Yes | Yes | Good |
| 34 | Mao2024 | Yes | No | Yes | Yes | NA | No | Yes | No | Poor |
| 35 | Mendolaro2024 | Yes | Yes | Yes | Yes | NA | No | No | No | Poor |
| 36 | Tanida2023 | Yes | Yes | Yes | No | NA | No | Yes | Yes | Good |
| 37 | Hilton2023 | Yes | Yes | No | No | NA | No | NA | No | Poor |
| 38 | Dos Santos2023 | Yes | Yes | Yes | Yes | NA | No | Yes | Yes | Good |
| 39 | Van Eycken2023 | Yes | Yes | Yes | No | NA | No | Yes | Yes | Good |
| 40 | Kooybaran2022 | Yes | Yes | No | No | NA | No | Yes | Yes | Fair |
| 41 | Colina 2025 | Yes | Yes | Yes | Yes | NA | No | NA | Yes | Good |
| 42 | Korbl 2022 | Yes | Yes | No | No | NA | No | NA | Yes | Fair |
| 43 | Zhang2023 | Yes | Yes | Yes | No | NA | No | Yes | Yes | Good |
| 44 | Nousari2021 | Yes | Yes | Yes | Yes | NA | No | Yes | Yes | Good |
| 45 | Melboucy-Belkhir 2018 | Yes | Yes | Yes | No | NA | No | Yes | Yes | Good |
| 46 | Chou2023 | Yes | Yes | Yes | No | No | NA | Yes | Yes | Fair |
| 47 | Jiang2021 | Yes | Yes | Yes | No | No | NA | NA | Yes | Good |
| 48 | Gowda2020 | Yes | Yes | No | NA | No | NA | NA | Yes | Fair |
| 49 | Thebo2019 | Yes | Yes | Yes | No | No | NA | Yes | Yes | Good |
| 50 | Sakoda2017 | Yes | Yes | Yes | No | Yes | NA | Yes | Yes | Good |
| 51 | Chatterjee2015 | Yes | No | No | No | No | NA | Yes | No | Poor |
| 52 | Orfaly 2021 | NA | Yes | Yes | No | NA | No | Yes | Yes | Good |
| 53 | Ramos 2024 | Yes | Yes | Yes | No | NA | No | Yes | Yes | Good |


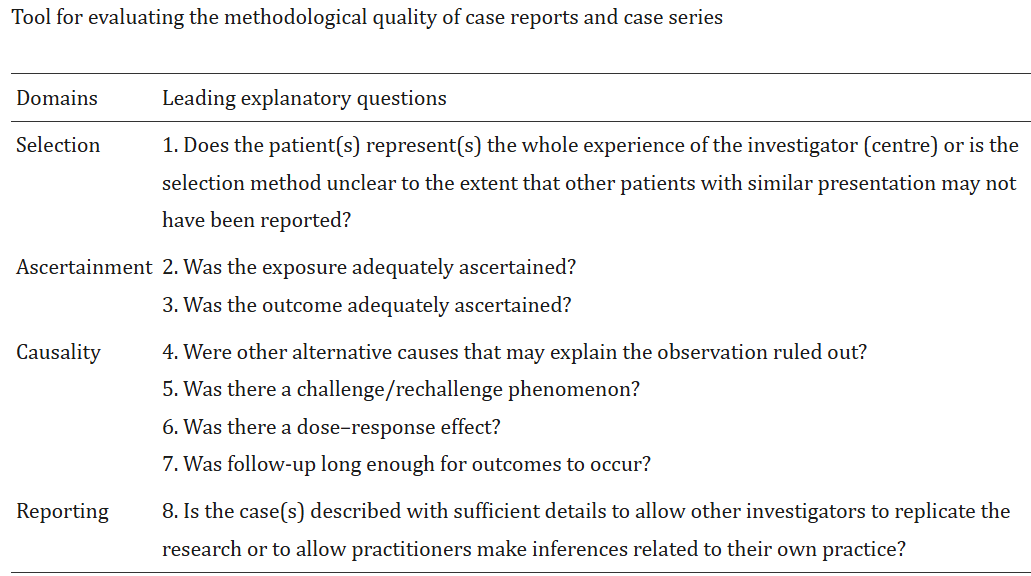


Quality Assessment Tool for Observational Cohort and Cross-Sectional Studies for Lee et al. 2022

| Criteria | Yes | No | Other (CD, NR, NA)* |
| --- | --- | --- | --- |
| 1. Was the research question or objective in this paper clearly stated? | + |  |  |
| 2. Was the study population clearly specified and defined? | + |  |  |
| 3. Was the participation rate of eligible persons at least 50%? |  | + |  |
| 4. Were all the subjects selected or recruited from the same or similar populations (including the same time period)? Were inclusion and exclusion criteria for being in the study prespecified and applied uniformly to all participants? | + |  |  |
| 5. Was a sample size justification, power description, or variance and effect estimates provided? | + |  |  |
| 6. For the analyses in this paper, were the exposure(s) of interest measured prior to the outcome(s) being measured? | + |  |  |
| 7. Was the timeframe sufficient so that one could reasonably expect to see an association between exposure and outcome if it existed? | + |  |  |
| 8. For exposures that can vary in amount or level, did the study examine different levels of the exposure as related to the outcome (e.g., categories of exposure, or exposure measured as continuous variable)? | + |  |  |
| 9. Were the exposure measures (independent variables) clearly defined, valid, reliable, and implemented consistently across all study participants? | + |  |  |
| 10. Was the exposure(s) assessed more than once over time? |  | + |  |
| 11. Were the outcome measures (dependent variables) clearly defined, valid, reliable, and implemented consistently across all study participants? | + |  |  |
| 12. Were the outcome assessors blinded to the exposure status of participants? |  | + |  |
| 13. Was loss to follow-up after baseline 20% or less? |  |  | + |
| 14. Were key potential confounding variables measured and adjusted statistically for their impact on the relationship between exposure(s) and outcome(s)? | + |  |  |

Quality Rating: Good
