# Supplementary material for: JAK Inhibitors for Treatment of Pyoderma Gangrenosum and Sweet Syndrome: A Systematic Review of Published Case Reports
Source: Dermatol Res Pract. 2026 Jul 18;2026:7086209. doi: 10.1155/drp/7086209 (PMC13379896; doi:10.1155/drp/7086209)
Supplement: Supplementary file 3 — Supporting Information 3 Supporting file 3 shows detailed tables of included studies. [file DRP-2026-7086209-s001.docx]

| Study | Age  Sex | Location  Past medical history | Disease duration  Biopsy | Previous Treatment | Treatment | Outcome  Adverse Effects |
| --- | --- | --- | --- | --- | --- | --- |
| Estrella et al.(1)  2025 | 54  Female | Lower extremity  PMH: UC | 4 months  No | Cephalexin, clindamycin, levofloxacin | abrocitinib  200 mg daily | Complete response within 4 months  Pain resolution  AEs: None |
| Yeo et al.(2)  2024 | 18  Male | Lower extremity, upper extremity, face  PMH: TA, HTN | 6 weeks  Yes | Fusidic acid cream, flucloxacillin, systemic and topical CSs, tocilizumab, leflunomide, IVIG, tacrolimus 0.1% ointment | abrocitinib  100 mg daily | Partial response after a month, treatment ceased due to side effects  AEs: acneiform eruption |
| Chen et al.(3)  2024 | 16  Male | Perianal area  PMH: None | 1 year  Yes | Doxycycline, Isotretinoin, CSs, CYA | abrocitinib  100 mg daily  16 weeks  + CYA | Partial response after 4 weeks  AEs: None |
| Wang et al.(4)  2024 | 44  Female | Lower extremity, trunk  PMH: NA | NA  Yes | None | Baricitinib  4 mg daily | Complete response within 6 months.  AEs: NA |
| Ito et al.(5)  2024 | 70  Female | Peristomal  PMH: RA, B/D | NA  No | CSs, colchicine, CYA, ADM, salazosulfapyridine | Baricitinib  Dosage NA  Switched to upadacitinib | Partial response within 9 weeks, without recurrence after 3 years  AEs: Baricitinib decreased renal function |
| Grisé et al.(6)  2024 | 82  Female | Lower extremity  PMH: SCC | 4 months  Yes | Mupirocin ointment, doxycycline, clobetasol ointment | Baricitinib 2mg daily (4 weeks) then 4 mg daily for 12 weeks | Complete response within 16 weeks  AEs: NA |
| Choe et al.(7)  2024 | 58  Male | Lower extremity, genital area  PMH: HTN, DM | 9 months  No | Clotrimazole cream, acyclovir, dapsone, cephalexin, ketoconazole cream, CYA, INX, tacrolimus ointment, silver sulfadazine, doxycycline | Baricitinib  (Dosage NA) | Partial response within 6 months.  AEs: NA |
| Bechard et. al(8)  2024 | 78  Female | Lower extremity, upper extremity  PMH: Metastatic ovarian carcinoma, PE, DLD, GERD | 6 months  Yes | CSs, Colchicine, Paclitaxel, IVIg, AB | Baricitinib  4mg daily  12weeks  +  CSs | Partial response within 12 weeks.  The lower extremity ulcer recurred 4 weeks after discontinuation of Baricitinib.  AEs: NA |
| Kim et. al(9)  2023 | 53  Male | Genital area  PMH: DM, Chronic B hepatitis, HCC | New onset  Yes | Atezolizumab, Bevacizumab, CSs, CYA, IVIg, AZA | Baricitinib  4 mg daily  +CSs | Partial response within 2 weeks.  Pain resolution  AEs: None |
| Castro et al.(10)  2023 | 73  Male | Upper extremity  PMH: Metabolic Syndrome, Inactive FMF | New onset  No | CSs | Baricitinib  2 mg BD (2 weeks) tapered to daily (5 weeks) | Complete response after 3 weeks.  AEs: None |
| Sitaru et al.(11)  2022 | 85  Female | Lower extremity  PMH: RA | 2 years  Yes | IVIg, MTX, Leflunomid, INX, MMF, CSs | Baricitinib  2 mg daily  1 year  + IVIg, CSs | Partial response within 2 months  AEs: None |
| Scheinberg et al.(12)  2021 | 71  Female | Scalp  PMH: IgA multiple myeloma,Pulmonary TB, PG | 18 years  Yes | CYA, INX, Clofazimine, Doxycycline, Intralesional Triamcinolone injections, Valacyclovir, Pregabalin, Dapsone | Baricitinib  4 mg daily | Complete response after 5 weeks  AEs: None |
|  | 59  Female | Lower extremity  PMH: RA, PG | 3 years  Yes | CSs, MTX | Baricitinib  4 mg daily  3 months | Partial response within 3 months  AEs: None |
| Shibuta et al. (13)  2025 | 57  Male | Trunk  PMH: hyperuricemia, glaucoma, JAK2V617F+ myelofibrosis | 1 month  Yes | CSs, ADM | Ruxolitinib 10 mg daily, then increased to 40 mg  +ADM, CSs | Partial response, and the patient died 22 months after receiving the drug due to myelofibrosis progression.  AEs: NA |
| Nasifoglu et al. (14)  2018 | 64  Female | Lower extremity  PMH: PV, PG, HTN, MS, Osteoporosis | 6 years  Yes | CSs, AZA, CYA, MMF, Anakinra, Phlebotomies, Hydroxyurea | Ruxolitinib  NA  3 years | Complete response within 10 weeks.  AEs: anemia |
| Shanmugam et al.(15)  2013 | 63  Male | Lower extremity  PMH: PV, colon cancer | 1 year  Yes | CSs, MMF, IVIg, pentoxifylline, SSZ, MTX, Warfarin, Heparin, Hydroxyurea, TNF-α inhibition | Ruxolitinib  10 mg/BD  18 months  + Hydroxyurea, MTX | Partial response within 5 weeks.  The patient died 18 months after treatment initiation due to cerebral hemorrhage  AEs: None |
| Xiao et al. (16)  2024 | 41  Male | Perianal area  PMH: None | 6 years  Yes | CSs, CYA, thalidomide, INX, SEC | Tofacitinib  10 mg daily | Complete response within 1.5 years,  AEs: None |
| Narula et al.(17)  2024 | 20  Female | Lower extremity  PMH: UC | 10 days  NA | CSs, CYA, MEZ | Tofacitinib 5 mg BD for the first week then 5 mg TDS for 4 weeks | Complete response after a month  AEs: NA |
| köken avşar et al.(18)  2024 | 41  Female | Lower extremity  PMH: UC, Bilateral knee arthritis | 23 years  Yes | SSZ, Diclofenac, CYA, INX, MTX, ADM, Certolizumab Pegol | Tofacitinib  10 mg daily  Two years  + MTX | Complete response after 15 months.  AEs: None |
| Castro et al.(10)  2023 | 89  Female | Lower extremity  PMH: None | 3 months  Yes | None | Tofacitinib  5 mg BD  8 months | Complete response within 4 months  AEs: None |
| Bhowmick et al.(19)  2023 | 22  Female | Lower extremity, upper extremity  PMH: Type IIb TA | 1 year  Yes | MTX, CSs, Colchicine, Tocilizumab, Cefepime, clindamycin, INX, Isoniazid, Rifampicin | Tofacitinib  5 mg/BD  13 months  +CSs | Complete response within 12 months.  AEs: None |
| Sathyanarayana et al.(20)  2023 | 70  Female | Lower extremity  PMH: None | 3 months  Yes | CSs, MTX | Tofacitinib  5 mg BD  24 weeks | Complete response within 12 weeks  AEs: None |
|  | 43  Male | Lower extremity  PMH: None | 1 month  Yes | TMP/SMX | Tofacitinib  5 mg BD  24 weeks | Partial response within 24 weeks.  AEs: None |
|  | 52  Female | Lower extremity  PMH: RA | 1 year  Yes | MTX | Tofacitinib  5 mg/BD  24 weeks  +MTX | Partial response within 24 weeks  AEs: None |
| Bhadresha et al.(21)  2022 | 41  Female | Trunk  PMH: Raynaud phenomenon, RA, Furunculosis, HS | 14 months  No | MTX, HCQ, Certolizumab Pegol, Rifampicin, clindamycin, AB, Doxycycline, systemic and topical CSs | Tofacitinib  5 mg/BD  NA  +CYA | Complete response within 3 months  AEs: None |
| Lee et al.(22)  2022  4 patients | NA  NA | Lower extremity, perianal area, NA in one patient  PMH: CD | NA  NA | Biologic therapies | Tofacitinib 10 mg BD for 8 weeks, then 5 mg BD  +Biologic Therapies | Partial response for all (one month and two months for two of them)  AEs: None |
| Orfaly et al.(23)  2021 | 41  Female | Lower extremity  PMH: MCTD, IBD | 7 years  No | CYA, Doxycycline, Hyperbaric O2, INX, MMF, MTX | Tofacitinib  10 mg daily  25 weeks  +IVIg, CSs | Partial response within 25 weeks  AEs: None |
|  | 58  Female | Lower extremity  PMH: RA | 3 years  No | ADM, CYA, ETC, Golimumab, INX, IVIg, KI, Minocycline, MTX, CSs | Tofacitinib  10 mg daily  21 weeks  +HCQ, CSs | Complete response within 21 weeks  AEs: None |
|  | 55  Female | Lower extremity  PMH: PsO, PsA | 20 months  No | CYA, Doxycycline, INX, MMF, CSs | Tofacitinib  10 mg daily  13 weeks  +IVIg | Complete response within 13 weeks  AEs: None |
|  | 70  Male | Lower extremity  PMH: UC | 5 months  No | CSs | Tofacitinib  10 mg-20mg daily  12 weeks  +CSs, Dapsone | Partial response within 12 weeks  AEs: None |
| Olavarría et al. (24)  2021 | 69  Female | Lower extremity, perianal area  PMH: Refractory PG, UC | 8 years  No | MEZ, AZA, INX, CYA, Vedolizumab, CSs | Tofacitinib  10 mg/BD  1 month  +CSs | Complete response within a month.  AEs: Arterial Hypertension |
| Sedano et al.(25)  2021 | 80  Female | Lower extremity  PMH: UC, PG, AA | 1 year  No | MTX, ADM, CSs, topical tacrolimus, Amino salicylates | Tofacitinib XL  11 mg daily  6 months | Partial response in 2 months  AEs: None |
| Choi et al.(26)  2020 | 64  Male | Generalized  PMH: Chronic rhinosinusitis, Cocaine use disorder | 10 months  No | CSs, dapsone, INX, AZA, CYA, ADM, Oral tacrolimus, RTX | Tofacitinib 5 mg BD and up-titrated to 10 mg BD  3 months  +CSs | Complete response after 3months  AEs: None |
| Gregory et al.(27)  2019 | NA  Female | Lower extremity, peristomal, genital area  PMH: Ulcerative Pancolitis, PG | 1 year  Yes | CSs, Dapsone, INX, AZA, MTX | Tofacitinib  10 mg/BD  3 weeks  + INX | Complete response within 3 weeks  AEs: NA |
| Kochar et al.(28)  2019 | 49  Female | Lower extremity  PMH: CD | New onset  Yes | Golimumab, CYA, UST | Tofacitinib  5 mg/BD  12 weeks  + None | Complete response within 12 weeks  AEs: None |
|  | 24  Male | Peristomal  PMH: CD | New onset    Yes | Vedolizumab, CSs | Tofacitinib  5 mg/BD  12 weeks  +Vedolizumab | completely response after 12 weeks  AEs: None |
|  | 34  Male | Lower extremity  PMH: PG, Perianal CD | 17 years  Yes | UST, CSs, Anti-TNF therapy | Tofacitinib 5 mg BD up to 10 mg BD more than a month  +CSs | Partial response within a month  AEs: None |
| Korytnikova et al.(29)  2025 | 26  Male | Lower extremity  PMH: none | 2.5 years  No | Systemic and topical CSs, dapsone, | Upadacitinib 15 mg daily  + CSs | Complete response within 34 weeks, with no recurrence at 12 months  AEs: NA |
| Taha et al. (30)  2025 | 47  Female | Lower extremity  PMH: RA, hypothyroidism | 8 years  Yes | CYA, CSs, MTX, MMF | Upadacitinib 15 mg daily, increased to 30 mg daily after 4 weeks, increased to 45 mg daily after 2 months | Complete response within 7 months  Pain resolution  AEs: none |
|  | 58  Female | Lower extremity, trunk  PMH: none | 1 year  Yes | MMF, systemic and topical CSs | Upadacitinib 15 mg daily | Complete response during 6 months  Pain resolution  AEs: body heaviness and fatigue |
| Patel et al.(31)  2025 | 44  Female | Lower extremity  PMH: CD | 2 years  Yes | CSs, INX, AZA, topical tacrolimus, AB, CYA | Upadacitinib  (Dosage NA) | Complete response within 6 months  AEs: NA |
| He et al.(32) 2025 | 36  Female | Lower extremity  PMH: SCZ, UC | 2 weeks  Yes | Systemic and topical AB, MEZ, ADM, CSs | Upadacitinib  45 mg daily  +CSs | Complete response within 7 weeks  AEs: none |
| Jimenez et al. (33)  2024 | 40  Female | Lower extremity  PMH: asthma, insulin resistance, UC, erythema nodosum, PsO, sacroiliitis | NA  NA | INX, UST, dapsone, leflunomide, cephalexin, mupirocin, TMP/SMX | Upadacitinib  45 mg daily | Complete response within 6 months, and no recurrence  AEs: NA |
| Ramos et al.(34)  2024 | 45  Male | Generalized  PMH: IBD | 1 year  NA | CSs, MTX, CYA, ADM | Upadacitinib 15 mg daily  26 weeks  +ADM, CSs | Complete response after 26 weeks  Pain resolution  AEs: none |
|  | 26  Female | Generalized  PMH: mammoplasty | 1 year  NA | CSs, MTX, CYA, ADM | Updacitinib  15 mg daily first month then 30 mg daily  16 weeks  + ADM and CSs | Partial response 16 weeks  Pain resolution  AEs: none |
|  | 62  Female | Lower extremity  PMH:RA, DLD, DM | 5 years  NA | CSs, MTX, CYA, ADM, golimumab | Upadacitinib 30 mg daily  16 weeks  +CSs | Partial response after 16 weeks  Pain resolution  AEs: none |
|  | 50  Male | Lower extremity  PMH: HTN, cocaine abuse | 10 years  NA | CSs, MTX, AZA, CYA, ADM, Certolizumab, INX | Upadacitinib 30 mg daily  12 weeks | Complete response after 12 weeks  Pain resolution  AEs: none |
| Zaher et al.(35)  2024 | 31  Male | Peristomal  PMH: UC, Human leukocyte antigen associated sacroiliitis with polyarthritis | NA  NA | CSs, CYA, dapsone, tofacitinib | Upadacitinib 30 mg daily | Complete response within 3 months  AEs: NA |
| Park et al.(36)  2024 | 62  Female | Lower extremity  PMH: UC | 1 year  Yes | Systemic and topical CSs, CYA, INX | Upadacitinib 30 mg daily, then 45 mg daily  8 weeks  +CSs | Partial response within 3 months  pain resolution  AEs: NA |
| Mao et al.(37)  2024 | 58  Female | Lower extremity  PMH: UC | 2 weeks  Yes | MEZ, INX, CSs | Upadacitinib  (Dosage NA) | complete response within 3 months  AEs: NA |
| Mendolaro  et al.(38)  2024 | 59  Female | Lower extremity, upper extremity  PMH: Colonic CD | New onset  No | CSs, INX | Upadacitinib  45 mg daily  6 weeks | complete response within 6 weeks  AEs: None |
| Tanida et al.(39)  2023 | 44  Female | Lower extremity  PMH: UC, Erythema Nodosum | New onset  Yes | Salazosulfapyridine, Loxoprofen, Sodium hydrate, CSs, MEZ, Cefazolin | Upadacitinib  45 mg daily  10 months  +GMA, CSs | Partial response after 10 weeks  AEs: Mild anemia |
| Hilton et al.(40)  2023 | 55  Female | Generalized  PMH: RA | New onset  Yes | ADM | Upadacitinib  +CSs, Dapsone, CP | Partial response (response time NA)  AEs: NA |
| Dos Santos et al.(41)  2023 | 45  Female | Lower extremity  PMH: RA | New onset  Yes | MTX, HCQ, CSs, AB, Dapsone | Upadacitinib  15 mg daily  6 weeks | Complete response after 6 weeks.  AEs: NA |
| Van Eycken et al.(42)  2023 | 65  Female | Lower extremity  PMH: SpA, PG | 6 years  Yes | MTX, INX, CSs, Tacrolimus, Systemic Minocycline, ECT, Doxycycline, Colchicine, Azithromycin, CYA, Apremilast, SEC | Upadacitinib  15 mg daily  24 weeks  + CSs | Complete response within 12 weeks  AEs: None |
| Kooybaran et al.(43)  2022 | 50  Female | Lower extremity  PMH: RA | 2 months    No | MTX, Leflunomide, Certolizumab Pegol, ADM, CSs, Pantoprazole, Vit D3, Rosuvastatin, Omega-3, IL-1 receptor antagonist Anakinra | Upadacitinib  15 mg daily  26 weeks  + CSs | Partial response within 2 weeks  Pain resolution  AEs: NA |

**Supplementary Table 1. JAK inhibitors usage in PG treatment**

UC: Ulcerative colitis, TA: Takayasu arteritis, CSs: Corticosteroids, IVIg: Intravenous immunoglobulin, CYA: Cyclosporine, RA: Rheumatoid arthritis, B/D: Behçet's disease, ADM: Adalimumab, SCC: Squamous Cell Carcinoma, HTN: Hypertension, DM: Diabetes Mellitus, INX: Infliximab, PE: Pulmonary Embolism, DLD: dyslipidemia, GERD: Gastroesophageal reflux disease, AB: Antibiotic, HCC: Hepatocellular carcinoma, FMF: Familial Mediterranean Fever, MTX: Methotrexate, MMF: Mycophenolate mofetil, TB: Tuberculosis, PG: Pyoderma gangrenosum, PV: Polycythemia vera, MS: Multiple sclerosis, SSZ: Sulfasalazine, SEC: secukinumab, UC: Ulcerative colitis, MEZ: mesalazine, HS: Hidradenitis suppurativa, HCQ: Hydroxychloroquine, ESR: Erythrocyte sedimentation rate, MCTD: Mixed connective tissue disease, PsO: Psoriasis, PsA: psoriatic arthritis, AA: Alopecia areata, CD: Crohn’s Disease, KI: Potassium iodide, UST: Ustekinumab, TNF: Tumor necrosis factor, SpA: Spondyloarthritis, ECT: Etanercept, vit: vitamin, TMP/SMX: Trimethoprim/sulfamethoxazole, RTX: rituximab, SCZ: schizophrenia, AZA: azathioprine, IBD: Inflammatory bowel disease, CP: Cyclophosphamide

| **Study** | **Age**  **Sex** | **Presentation**  **Disease duration** | **Medical history**  **+ Biopsy** | **Previous Treatments** | **Treatments** | **Outcome**  **Adverse Effects** |
| --- | --- | --- | --- | --- | --- | --- |
| Colina et al.(44)  2025 | 64  Female | Rapid onset of painful infiltrated plaques, high-spiking fever, neutrophilic leukocytosis, and systemic symptoms  4 years | RA  +Yes | HCQ, MTX, leflunomide, tocilizumab, CSs | Filgotinib  200 mg daily | Complete response  AEs: NA |
| Korbl et al.(45)  2022 | 55  Female | Multiple tender erythematous pseudovesicular plaques (over face, upper back and the dorsum of the right hand), fever  New onset | MGUS (IgGK) monoclonal  B cell lymphocytosis  +Yes | MTX, CYA, Dapsone, HCQ, Thalidomide, KI, CTX, Betamethasone, CSs, Colchicine, Dipropionate, Indomethacin, Anakinra | Ruxolitinib  20 mg BD  one month  +CSs | Complete response within 1 month  AEs: NA |
| Zhang et al.(46)  2023 | 50  Female | Bilateral recurrent annular erythematous plaques on hands, progressive pain, stomach ache  3 years | Chronic gastritis  +Yes | CSs | Baricitinib  2 mg daily  2 months  +CSs | Complete response within 1 month  AEs: None |
| Nousari et al.(47)  2021 | 59  Female | Mildly painful non-scaly, erythematous and edematous papules and plaques on her mid-chest, malaise, low-grade fever, polyarthralgia, and small joint stiffness  10 months | RA  +Yes | CSs, Golimumab,  Leflunomide, Diclofenac, INX, RTX, MTX, Abatacept, Tocilizumab | Baricitinib  2 mg daily  10 months  +None | Complete response within 1 month  AEs: None |
| Melboucy-Belkhir et al.(48)  2018 | 66  Male | Long-lasting fever, erythematous and painful skin lesions on buttocks and lower limbs, flags of ears and fingers  New onset | JAK-2 positive myeloproliferative disease (essential thrombocythemia with MF)  +Yes | CSs, Colchicine, anakinra, hydroxyurea | Ruxolitinib 10 mg BD  18 months  +CSs | Complete response during 18 months; However, disease recurred parallel to progression to secondary AML, and the patient died of respiratory distress syndrome related to pulmonary leukostasis  AEs: NA |

**Supplementary Table 2. JAK inhibitors usage for Sweet’s syndrome treatment.**

RA: Rheumatoid arthritis, HCQ: Hydroxychloroquine, MTX: Methotrexate, CSs: Corticosteroids, MGUS: Monoclonal gammopathy of undetermined significance, CYA: Cyclosporine, KI: Potassium iodide, CTX: Cyclophosphamide, BD: Twice a day, INX: Infliximab, RTX: rituximab, MF: myelofibrosis, AML: acute myeloid leukemia

| **Study** | **Age**  **Sex** | **Presentation**  **Disease duration** | **Medical history**  **+ Biopsy** | **Previous Treatments** | **JAK inhibitor/ Reason for usage** | **Time to Onset** | **Outcome** |
| --- | --- | --- | --- | --- | --- | --- | --- |
| Chou et. al.(49)  2023 | 69  Male | Fever, dyspnea, malaise, anorexia, and cough, low O2 saturation, tender erythematous papule on his right wrist, which increased in size over several days  New onset | JAK2V617F+ post-PV MF  +Yes | IV AB, CSs, Dapsone | Ruxolitinib+  CSs, Dapsone/  controlling MF symptoms | NA | Treatment with methylprednisolone demonstrated rapid resolution of skin lesions and dyspnea |
| Jiang et al.(50)    2021 | 77  Male | Abdominal pain, coryzal symptoms, well-defined, erythematous and painful lesions with a violaceous edge multiple lesions on the surgical site of the lower abdomen, eventually forming superficial erosions, on the glans penis with a pseudovesicular, edematous appearance  New onset. | Transfusion-dependent MF,  terminal ileitis  +Yes | AB, Danazol, IV AB, G-CSF | Ruxolitinib  10mg daily+  Danazol, CSs/  MF symptoms management | 3 weeks | Lesions resolved after a course of systemic and topical CSs, without withdrawal of ruxolitinib |
| Gowda et. al.(51)  2020 | 66  Female | Fevers, chills, nausea, cough, bruise-like wound with erythema, warmth, edema and pain in the right leg  New onset | Hypothyroidism, Sjogren syndrome, post-ET MF, AML  +Yes | IV AB, CSs, IVIg, | Ruxolitinib (dosage NA)/  Ruxolitinib was received as chemotherapy | NA | Treatment with CSs and IVIG resulted in rapid improvement of symptoms |
| Thebo et. al.(52)  2019 | 46  Male | Right shin lesion, fatigue, dyspnea on exertion, and weight loss  Two months | MF  +Yes | AB, Decitabine, Vancomycin, Piperacillin, Tazobactam, Voriconazole | Ruxolitnib  20 mg BD then 20mg daily  + Decitabine/  improvement of splenomegaly-associated symptoms (abdominal discomfort) | 3 months | Treatment with CSs resulted in rapid resolution of fever, skin lesions and pain |
| Sakoda et. al.(53)  2017 | 59  Male | Weight loss, hepatosplenomegaly,  painful erythematous plaque on his right buttock with a high fever, on day 16, a similar painful erythematous lesion on other side  New onset | Post-ET MF, GVHD  +Yes | Meropenem, Daptomycin, CSs, Fludarabine, Busulfan, Tacrolimus, MTX, G-CSF | Ruxolitinib  30 mg daily  9 weeks+ PSL/  To improve the splenomegaly | 7 weeks | Ruxolitinib was withdrawn and both fever and skin lesions resolved |
| Chatterjee et. al.(54)  2015 | 77  Female | General deterioration in well-being, multiple pustular lesions, and swelling of both hands, abdomen, back and legs  New onset | Post-ET MF  +Yes | AB, CSs, Dapsone | Ruxolitinib  NA  12 months+NA/  Controlling the constitutional symptoms and splenomegaly | 18 months | Dapsone was started to control the symptoms, but patient expired without resolution |

**Supplementary Table 3. JAK inhibitor-associated Sweet’s syndrome.**

PV: Polycythemia vera, MF: Myelofibrosis, AB: Antibiotics, CSs: Corticosteroids, G-CSF: Granulocyte colony-stimulating factor, AML: acute myeloid leukemia, IVIg: Intravenous immunoglobulin, SS: Sweet syndrome, BD: Twice a day, GVHD: Graft-versus-Host Disease, MTX: Methotrexate, post-ET: post-essential thrombocythemia myelofibrosis, PSL: systemic prednisolone

**References**

1. Estrella MME, Verallo-Rowell VM. Pyoderma gangrenosum treated with oral abrocitinib in a 54-year-old woman: A case report. JAAD Case Reports. 2025;60:4-6.

2. Yeo TF, Labbouz S, Lawrance N, Sitaraaman HB, Tattersall RS, Cork MJ. Refractory pyoderma gangrenosum in Caucasian adolescent with Takayasu arteritis and life‐threatening infections. JEADV Clinical Practice. 2025;4(1):234-9.

3. Chen P, Liang J, Li C, Li Q, Liu W, Zhu J, et al. Abrocitinib as a novel treatment for multiple skin disorders: 3 case reports and a scoping review. Clinical, Cosmetic and Investigational Dermatology. 2024:35-40.

4. Wang Z, Li T, Gong L, Song Z, Piao Y. Successful treatment of multiple site involvement pyoderma gangrenosum with baricitinib. International Journal of Dermatology. 2024;63(10).

5. Ito H, Noda K, Saruta M, Kurosaka D. Case report: Peristomal pyoderma gangrenosum complicated by rheumatoid arthritis and Behçet's disease successfully treated with baricitinib. International Journal of Rheumatic Diseases. 2024;27(7).

6. Grisé A, Valere L-C, Weinstein D, Sami N. Janus kinase inhibitors in the treatment of pyoderma gangrenosum: case report and review. Archives of Dermatological Research. 2024;316(6):238.

7. Choe SI, Shettig A, Kody S, Vague M, Hoff A, Rios-Duarte JA, Ortega-Loayza AG. Pyoderma gangrenosum of the genitalia, anus, and perineum: two case reports and a review of published cases. Sexually Transmitted Diseases. 2024;51(8):548-50.

8. Bechard K, Gniadecki R. Use of Baricitinib in a patient with treatment-resistant pyoderma gangrenosum. SAGE Open Medical Case Reports. 2024;12:2050313X241235444.

9. Kim HS, Kwon JE, Park YJ. Atezolizumab plus bevacizumab-induced recalcitrant pyoderma gangrenosum treated with baricitinib: a case report. Acta Dermato-Venereologica. 2023;103:9646.

10. Castro LG. JAK inhibitors: a novel, safe, and efficacious therapy for pyoderma gangrenosum. International Journal of Dermatology. 2023;62(8):1088-93.

11. Sitaru S, Biedermann T, Lauffer F. Successful treatment of pyoderma gangrenosum with Janus kinase 1/2 inhibition. JEADV Clinical Practice. 2022;1(4):420-3.

12. Scheinberg M, Machado LA, Castro LGM, Ferreira SB, Michalany N. Successful treatment of ulcerated pyoderma gangrenosum with baricitinib, a novel JAK inhibitor. Journal of translational autoimmunity. 2021;4:100099.

13. Shibuta K, Hayama K, Miura K, Fujita H. Successful treatment of pyoderma gangrenosum complicated by JAK2V617F mutation-positive myelofibrosis with adalimumab and systemic steroid. Journal of Cutaneous Immunology and Allergy. 2025;8:14079.

14. Nasifoglu S, Heinrich B, Welzel J. Successful therapy for pyoderma gangrenosum with a Janus kinase 2 inhibitor. British Journal of Dermatology. 2018;179(2):504-5.

15. Shanmugam VK, McNish S, Shara N, Hubley KJ, Kallakury B, Dunning DM, et al. Chronic leg ulceration associated with polycythemia vera responding to ruxolitinib (Jakafi®). The Journal of Foot and Ankle Surgery. 2013;52(6):781-5.

16. Xiao Y, Liao S, Hu D, Li R, Tu P, Wang X, Zhong S. Refractory Giant Perianal Pyoderma Gangrenosum Successfully Treated with Tofacitinib. Indian Journal of Dermatology. 2024;69(4):343-4.

17. Narula S, Chanana K, Thole A, Sardana K, Muddebihal A. A Case of Multifocal Pyoderma Gangrenosum with Cyclosporine Induced Neurotoxicity and Its Exquisite Response to Tofacitinib. Indian Dermatology Online Journal. 2025;16(3):455-6.

18. Köken Avşar A, Demirci Yıldırım T, Sarı İ. Tofacitinib therapy for severe pyoderma gangrenosum in a patient with enteropathic arthritis: a case-based review. Rheumatology International. 2024;44(10):2227-37.

19. Bhowmick K, Roongta R, Dey S, Dey S, Mondal S, Sinhamahapatra P, et al. Refractory Takayasu arteritis with recurrent pyoderma gangrenosum: a therapeutic challenge with case-based review. Clinical Rheumatology. 2023;42(5):1469-77.

20. Sathyanarayana VA, Roy D, Nagaraju B, Rao VK. Tofacitinib in pyoderma gangrenosum–A case series. International Journal of Rheumatic Diseases. 2024;27(1):e14810.

21. Bhadresha S, Connolly A, Galloway J, Walsh S. A noduloulcerative plaque in a patient with rheumatoid arthritis. Clinical & Experimental Dermatology. 2022;47(1).

22. Lee SD, Singla A, Harper J, Barahimi M, Jacobs J, Kamp KJ, Clark-Snustad KD. Safety and efficacy of tofacitinib in combination with biologic therapy for refractory Crohn’s disease. Inflammatory Bowel Diseases. 2022;28(2):309-13.

23. Orfaly V, Kovalenko I, Tolkachjov S, Ortega‐Loayza A, Nunley J. Tofacitinib for the treatment of refractory pyoderma gangrenosum. Clinical and Experimental Dermatology. 2021;46(6):1082-5.

24. Olavarría PS, Iturria SR, Castillejo ÓN. Tofacitinib, a useful option for the treatment of pyoderma gangrenosum in an ulcerative colitis patient. Rev Esp De Enfermedades Dig. 2021;113:733-4.

25. Sedano R, Jairath V. Tofacitinib for the treatment of three immune-mediated conditions in one patient: ulcerative colitis, pyoderma gangrenosum, and alopecia areata. Inflammatory Bowel Diseases. 2021;27(5):e65-e.

26. Choi AW, Abuav R, Rabizadeh SM, Ansari R, Marsch AF. Recalcitrant and severe pyoderma gangrenosum attributable to levamisole-adulterated cocaine and treated successfully with oral tofacitinib. JAAD Case Reports. 2020;6(9):939-41.

27. Gregory MH, Ciorba MA, Deepak P, Christophi GP. Successful treatment of pyoderma gangrenosum with concomitant tofacitinib and infliximab. Inflammatory bowel diseases. 2019;25(7):e87-e8.

28. Kochar B, Herfarth N, Mamie C, Navarini AA, Scharl M, Herfarth HH. Tofacitinib for the treatment of pyoderma gangrenosum. Clinical Gastroenterology and Hepatology. 2019;17(5):991-3.

29. Korytnikova E, Halasz CL, Sangeorzan E. Upadacitinib for Pyoderma Gangrenosum: A Case Report and Review of Emerging Evidence. SKIN The Journal of Cutaneous Medicine. 2025;9(4):2506-12.

30. Taha MR, Nguyen HP, Tyring SK. Upadacitinib monotherapy for treatment of pyoderma gangrenosum. American Journal of Medicine Open. 2025:100112.

31. Patel M, Woo P-n, Ahmed F. BI24 A case of refractory pyoderma gangrenosum successfully treated with upadacitinib. British Journal of Dermatology. 2025;193(Supplement_1):ljaf085. 433.

32. He S-D, Tian Y. Upadacitinib for ulcerative colitis and pyoderma gangrenosum in a patient with schizophrenia on long-term risperidone: A case report. World Journal of Gastroenterology. 2025;31(20):104038.

33. Jimenez PMP, Tabib S, Abbott B, Melmed G. Successful Outcome Treating Pyoderma Gangrenosum and Pouchitis With Upadacitinib. ACG Case Reports Journal. 2024;11(8):e01442.

34. Ramos FM, García-Ruíz R, Vázquez AA, Mercader-García P. Four-case Report of Upadacitinib as an Alternative Treatment for Patients With Recalcitrant Pyoderma Gangrenosum. Actas dermo-sifiliograficas. 2024;115(10):1020-3.

35. Zaher A, Castillo M. S3860 Successful Treatment of Refractory Peristomal Pyoderma Gangrenosum in an Ulcerative Colitis Patient With Upadacitinib: A Case Report. Official journal of the American College of Gastroenterology| ACG. 2024;119(10S):S2521-S2.

36. Park S, St Pierre J, Onajin O, Rubin DT. Successful Treatment of Severe Pyoderma Gangrenosum and Ulcerative Colitis With Upadacitinib. ACG Case Reports Journal. 2024;11(10):e01531.

37. Mao X-Y, Yang Y-Y, Tian L. A Win-Win Solution: Remarkable Reversal of Foot Necrosis in Ulcerative Colitis. Gastroenterology. 2025;169(1):30-3.

38. Mendolaro M, Morello E, Salacone P, Rocca R. A case of refractory severe pyoderma gangrenosum successfully treated with upadacitinib. Digestive and Liver Disease. 2024;56(7):1248.

39. Tanida S, Kubo R, Yoshii S, Takahama T, Sasoh S, Kubota Y, et al. Upadacitinib plus intensive granulocyte and monocyte adsorptive apheresis for ulcerative colitis achieved ulcer healing for pyoderma gangrenosum. Journal of Clinical Medicine Research. 2023;15(10-11):446.

40. Hilton B, Cleaver D, Cleaver L. 44360 adalimumab paradoxically causing pyoderma gangrenosum. Journal of the American Academy of Dermatology. 2023;89(3):AB13.

41. Dos Santos MR, Ianhez M, Ribeiro BN, de Queiroz BB, Miot HA. Refractory pyoderma gangrenosum associated with rheumatoid arthritis successfully treated with upadacitinib. Comments on:" JAK inhibitors: a novel, safe, and efficacious therapy for pyoderma gangrenosum". International Journal of Dermatology. 2023;62(11).

42. Van Eycken L, Dens A-C, de Vlam K, Neerinckx B, De Haes P. Resolution of therapy-resistant pyoderma gangrenosum with upadacitinib. JAAD Case Reports. 2023;37:89-91.

43. Kooybaran NR, Korsten P, Schön MP, Mössner R. Response of rheumatoid arthritis‐associated pyoderma gangrenous to the JAK1 inhibitor upadacitinib. 2022.

44. Colina M, Barisani A, Gualandi A, Poli F, Campana G. Successful use of Filgotinib in the Treatment of Refractory Rheumatoid Arthritis-Associated Sweet Syndrome. Journal ISSN. 2025;2766:2276.

45. Korbl J, Smith A, Wood B, Chua H, Pepperell D, Kirupananther R, Donnelly A, editors. Refractory Sweet Syndrome Successfully Treated with Ruxolitinib. AUSTRALASIAN JOURNAL OF DERMATOLOGY; 2022: WILEY 111 RIVER ST, HOBOKEN 07030-5774, NJ USA.

46. Zhang H, Xia P, Wu N, Chen J, Liu Y. Neutrophilic dermatosis of the dorsal hands treated with baricitinib. Clinical and Experimental Dermatology. 2023;48(11):1274-6.

47. Nousari Y, Wu B, Valenzuela G. Successful use of baricitinib in the treatment of refractory rheumatoid arthritis‐associated Sweet syndrome. Clinical and Experimental Dermatology. 2021;46(7):1330-2.

48. Melboucy-Belkhir S, Brigant F, Khentache R, Bouketouche M, Garidi R, Brihaye B. Sweet syndrome successfully treated with ruxolitinib in JAK-2 positive myeloproliferative disorder. Int Arch Intern Med. 2017;2:8-10.

49. Chou C, Chatterjee AB. PULMONARY SWEET SYNDROME IN A PATIENT WITH MYELOFIBROSIS. Chest. 2023;164(4):A5545-A6.

50. Jiang M, Tran AK, Marshman G. A neutrophilic dermatosis following treatment of myelofibrosis with ruxolitinib: An emerging phenomenon? Australasian Journal of Dermatology. 2021;62(4).

51. Gowda A, Christensen L, Polly S, Barlev D. Necrotizing neutrophilic dermatosis: a diagnostic challenge with a need for multi-disciplinary recognition, a case report. Annals of Medicine and Surgery. 2020;57:299-302.

52. Thebo U, Tummala S, Nassereddine S, Haroun F. An atypical presentation of Sweet’s syndrome in a myelofibrosis patient. BMJ Case Reports CP. 2019;12(3):e228076.

53. Sakoda T, Kanamitsu Y, Mori Y, Sasaki K, Yonemitsu E, Nagae K, et al. Recurrent Subcutaneous Sweet's Disease in a myelofibrosis patient treated with ruxolitinib before allogeneic stem cell transplantation. Internal Medicine. 2017;56(18):2481-5.

54. Chatterjee B, Rqieh U, Greaves P, Piras D, Firth J, Saja K. Sweet syndrome as terminal event in ruxolitinib-treated myelofibrosis. British Journal of Haematology. 2015;169(3).
